# Identification and Chronological Analysis of Genomic Signatures in Influenza A Viruses

**DOI:** 10.1371/journal.pone.0084638

**Published:** 2014-01-08

**Authors:** Yuh-Jyh Hu, Po-Chin Tu, Chun-Sheng Lin, Szu-Ting Guo

**Affiliations:** 1 Institute of Biomedical Engineering, National Chiao Tung University, Hsinchu, Taiwan; 2 Department of Computer Science, National Chiao Tung University, Hsinchu, Taiwan; National Institute for Viral Disease Control and Prevention, CDC, China, China

## Abstract

An increase in the availability of data on the influenza A viruses (IAV) has enabled the identification of the potential determinants of IAV host specificity using computational approaches. In this study, we proposed an alternative approach, based on the adjusted Rand index (ARI), for the evaluation of genomic signatures of IAVs and their ability to distinguish hosts they infected. Our experiments showed that the host-specific signatures identified using the ARI were more characteristic of their hosts than those identified using previous measures. Our results provided updates on the host-specific genomic signatures in the internal proteins of the IAV based on the sequence data as of February 2013 in the National Center for Biotechnology Information (NCBI). Unlike other approaches for signature recognition, our approach considered not only the ability of signatures to distinguish hosts (according to the ARI), but also the chronological relationships among proteins. We identified novel signatures that could be mapped to known functional domains, and introduced a chronological analysis to investigate the changes in host-specific genomic signatures over time. Our chronological analytical approach provided results on the adaptive variability of signatures, which correlated with previous studies’ findings, and indicated prospective adaptation trends that warrant further investigation.

## Introduction

Influenza A viruses (IAV) are members of the Orthomyxoviridae family, and are enveloped negative-stranded RNA viruses with a segmented genome [1]. The envelope of an IAV consists of 2 surface glycoproteins, HA and NA, and a small domain of the M2 protein, underlain by the matrix protein M1. The IAV have the capacity to evade host immune systems because of a wide variety of potential combinations of the 16 HA and 9 NA subtypes. Because of their vast genetic diversity and unique host range, IAV have caused recurrent annual epidemics and several major worldwide pandemics in human history.

The accumulation of point mutations during genome replication, and the reassortment of viral gene segments during mixed infections, promotes the evolution of influenza viruses [2]. Because the number of viral sequences is continuously increasing, investigators have developed computational methods to recognize and verify interspecies transmission candidate determinants at the sequence level, despite the absence of specific knowledge on the antigenic properties of the viruses being investigated. For example, a number of large-scale phylogenetic and sequence alignment analyses have suggested that the IAV ribonucleoprotein (RNP) genes have evolved into divergent host-associated lineages, and that selected amino acids at specific positions of each internal protein are characteristic of the species origin for the sequences [3]–[6].

The successful establishment of an influenza virus in a new host is rare because it is a multistep process that requires the efficient and effective transmission, replication, and adaptation of the virus. However, pandemics caused by widely circulating viruses with the potential to transmit to humans remain a threat [2]. The emergence and spread of novel IAV remain of major global concern; therefore, increased understanding of the host range is essential to maintain the efficacy of antiviral drugs and influenza vaccines. In addition to the analysis of the molecular mechanisms underlying host specificity, using in vitro systems and reverse genetics of influenza viruses, the analysis of a considerable amount of available viral sequence data provides a cost-effective approach for the identification of host-associated genomic signatures as host-range determinants.

In this study, we proposed an alternative measure for the evaluation of the host-specific characteristic sites in the IAV based on the adjusted Rand index (ARI) [7], and produced a novel catalogue of host-specific genomic signatures from the viral sequence data as of February 2013 in the National Center for Biotechnology Information (NCBI). In comparison with the sites identified from the same sequence data using measures such as entropy and mutual information (MI), the sites we identified have higher species specificity for the determination of the host range. Genomic signatures can change because of point mutations or interspecies reassortments; therefore, we also performed a chronological analysis of the genomic signatures. We divided the IAV data into chronological groups according to the time of their discovery, and identified the genomic signatures in each of the groups. These signatures were host-specific and time-specific. We analyzed the transitions of these signatures across various periods to evaluate the adaptation trends, and successfully identified several adaptation trends that correlated with the results by related studies. We also identified additional adaptation patterns that warrant further investigation.

## Materials and Methods

**Figure 1:**
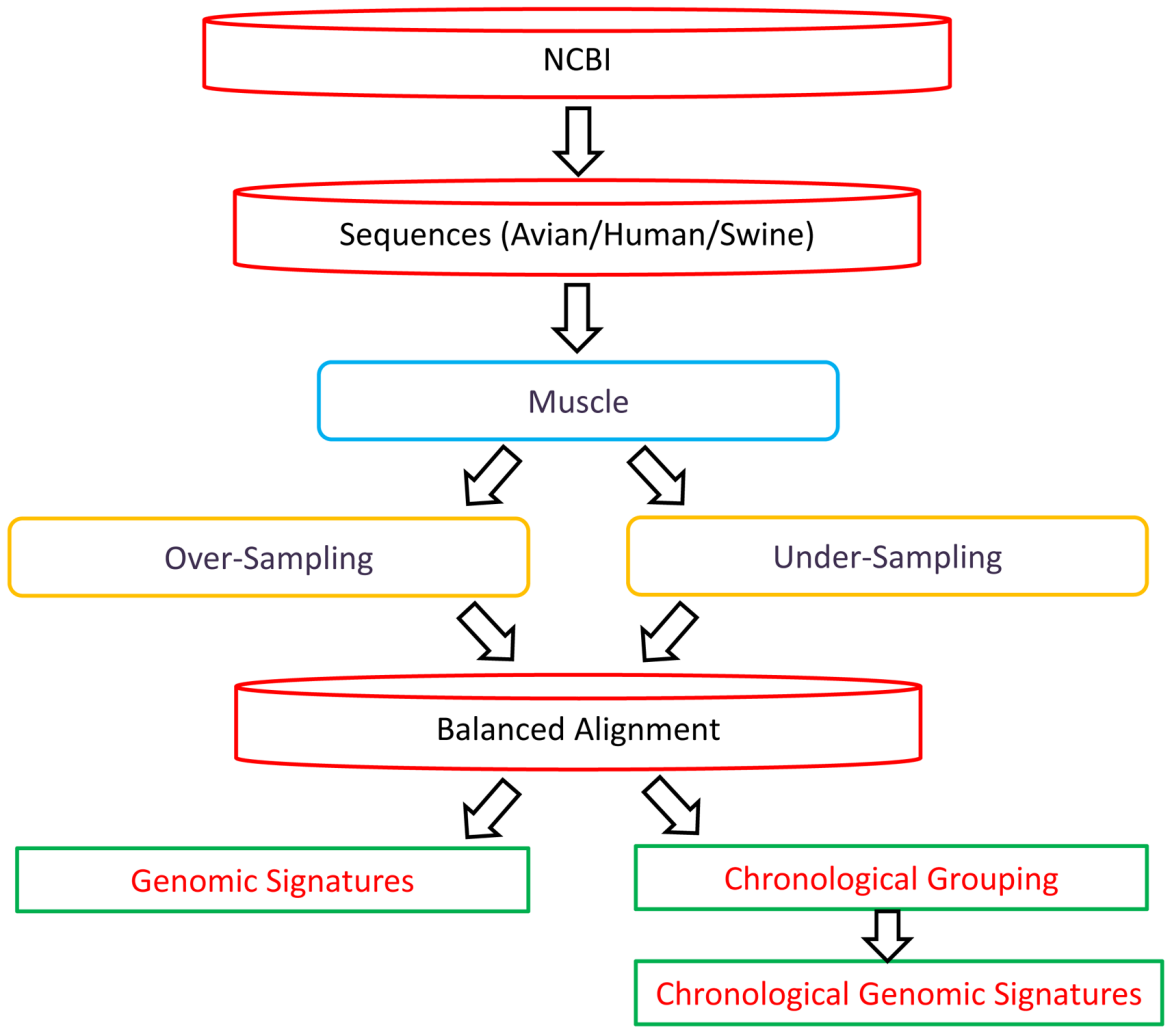
Process flow of genomic signature identification and chronological analysis.

We show the process flow of this study’s approach for host-specific genomic signature identification and chronological analysis in Figure 1. All available IAV sequences in the NCBI in February, 2013 were downloaded. The data were postprocessed by removing the redundant sequences and sequences missing quality annotations. The HA and NA proteins were excluded because of their genetic diversity, which impedes the production of satisfactory alignments. The influenza proteins were classified into 3 groups according to the type of host: avian, human, and swine (Table 1). The multiple sequence alignment tools ClustalW [8] and MUSCLE [9] were applied to the IAV protein sequences, and the alignments were then analyzed to identify the characteristic sites as potential signatures to distinguish different host-restricted IAV proteins.

**Table 1:** Number of influenza A sequences in study (1902–2013).

Host\Protein	PB2	PB1	PA	NP	M1	M2	NS1	NS2	PB1-F2
**Avian**	4207	3823	4052	2657	1144	1406	2624	1245	2215
**Human**	3025	2713	2698	1768	992	1478	1960	789	780
**Swine**	1129	1076	1109	985	625	834	951	579	379

Imbalance in the number of sequences can affect the identification of genomic signatures. To alleviate bias, two random sampling strategies were adopted to balance the data size in an alignment: undersampling and oversampling. For example, the number of avian PA records is 4052, which is substantially larger than 2698, the number of human PA records. In an alignment of all sequences, avian PA clearly dominates human PA, which could bias the calculation of the significance of the characteristic sites toward the avian PA. Using undersampling, 2698 avian PA sequences were randomly sampled without replacement, and combined with the 2698 human PA sequences to form a balanced alignment with an identical number of avian PA and human PA. Using oversampling, 4052 human PA sequences were randomly sampled with replacement, and combined with the 4052 avian PA sequences to form a second balanced alignment. Each column (i.e., each site) in the balanced alignments produced by undersampling and oversampling was evaluated for its significance. Multiple undersampling and oversampling runs were performed to reduce variance in the calculations of significance, and the genomic signatures were identified according to the average significance of each site.

Previous large-scale studies have used several computational methods to evaluate the significance of host-specific genomic signatures. A study on the IAV sequences as of May 2009 in the NCBI used an entropy measure to identify 47 avian-human signatures in the proteins PB1, PB2, PA, NP, M1, M2, NS1, and NS2 [10]. Finkelstein et al. used statistical analyses of residue frequencies from pandemic and H5N1 influenza viruses to identify a catalogue of 32 persistent host markers [11]. A genome-wide association analysis by Miotto et al. applied MI to identify the characteristic sites in avian and human strains of the IAV [12]. Using methods based on phylogenetic models, Tamuri et al. identified amino acid sites with strong support of the selection constraints in avian and human viruses [13]. Entropy measures the degree of uncertainty of a variable, whereas MI examines the strength of an association between two variables. Our study proposed the use of the adjusted Rand index (ARI) [7], an extension of the Rand index [14], for the evaluation of the ability of characteristic sites to distinguish between different hosts.

A higher ARI value indicates greater agreement between the 2 partitions. If *P* is compared to the partition of the IAV protein sequences according to the host (e.g., avian vs. human), and *Q* is compared to the partition based on the amino acids in a particular column of the protein sequence alignment, a column with a high ARI value is a characteristic site of the host-specific protein sequences. Using 11 sequences for illustration, an alignment was partitioned into 2 subsets according to the host (Table 2). This partition was termed *P* = {(s_1_, s_2_, s_3_, s_4_, s_5_, s_6_), (s_7_, s_8_, s_9_, s_10_, s_11_)}. Based on the amino acids in Site 1, the sequences were partitioned into 4 subsets, denoted by *Q1* = {(s_1_), (s_2_, s_3_, s_4_, s_5_, s_6_), (s_7_, s_8_, s_9_), (s_10_, s_11_)}. Similarly, based on the amino acids in Site 2, the sequences were partitioned into 2 subsets, denoted by *Q2* = {(s_1_, s_7_, s_8_, s_9_), (s_2_, s_3_, s_4_, s_5_, s_6_, s_10_, s_11_)}. The first 2 subsets of *Q1* are exclusively avian, and the other 2 subsets are exclusively human, whereas the subsets of *Q2* are both mixed avain and human. Therefore partition *Q1* is more specific to the hosts than *Q2*. The ARI between *P* and *Q1* was 0.58, and the ARI between *P* and *Q2* was 0.13, which reflects the fact that Site 1 is preferable to Site 2. A study by Milligan and Cooper evaluated several different indices for the measurement of the agreement between 2 partitions, and recommended the ARI [15]. Therefore, in this study, the ARI was adopted for the evaluation of the host-specific characteristic sites in the IAV.

**Table 2:** An example of the ARI for host-restricted sites.

Host	Sequence	Site 1	Site 2
**Avian**	S_1_	P	P
	S_2_	Q	Q
	S_3_	Q	Q
	S_4_	Q	Q
	S_5_	Q	Q
	S_6_	Q	Q
**Human**	S_7_	C	P
	S_8_	C	P
	S_9_	C	P
	S_10_	L	Q
	S_11_	L	Q
**ARI**	–	0.58	0.13

To investigate the adaptation trends, the IAV sequences were partitioned into chronological groups according to the time of their discovery: 1902–1918, 1919–1957, 1958–1968, 1969–1977, 1978–2009, and 2010–2013. The IAV sequences identified during the different periods were aligned separately and balanced between different hosts using the described random sampling techniques. For each column (i.e., a site) in an alignment, the average ARI was calculated to measure its association with the host, and the characteristic sites were identified. The characteristic sites identified from different chronological groups were then compared to further investigate 2 types of adaptation trend: validity and identity.

Validity refers to a genomic signature identified in one period becoming a nonsignature in subsequent periods, or vice versa. A site is considered “valid” within a period if it is a host-specific signature within that period. When a valid site is no longer a signature within another period, it becomes an “invalid” site. An invalid site in one period can become valid within a different period if it is a host-specific site during that period. The purpose of the validity analysis was to examine the sites for changes in validity, caused by amino acid substitutions, over time.

Identity refers to the changes, or absence of changes, in the amino acid residues of a site that remains constantly valid. For example, in the 2009 H1N1 pandemic strains, one amino acid at position NP-100 mutated from V during the preepidemic period to I during the late period [16]. The identity analysis enabled the monitoring of the amino acid residue transitions on the characteristic sites over time.

## Results

### Identification of the Host-specific Genomic Signatures

In this study, we aligned the human IAV protein sequences in the NCBI between 1902 and 2013 with the avian and swine IAV protein sequences using the MUSCLE software [9]. After balancing the size differences between the different host groups using random sampling (see “Materials and Methods”), we calculated the average ARI for each site in the alignments.

In our analyses of genomic signatures, based on the ARI, we considered the top 20 sites, and selected only the higher-ranked sites that differed in their dominant amino acids as genomic signatures. We identified 129 avian-human and 77 swine-human genomic signatures in the internal proteins PB1, PB2, PA, NP, M1, M2, NS1, NS2, and PB1-F2, an alternative protein product of PB1. Table 3 shows the numbers of signatures in each protein. Tables 4 and 5 show the top-ranked characteristic sites with discriminating amino acid residues. We compared these signatures with those reported previously [10], [12], [13], [16], as shown in Tables 6 and 7. Several previously reported characteristic sites were unable to distinguish the protein sequences of different species in the more recent data used in our study. However, we discovered some novel genomic signatures. For example, in the NP protein, we eliminated positions 375 and 423 as avian-human signatures, but identified novel signatures at positions 351 and 353. In the swine group, we identified NP-16, 283, and 313 as novel swine-human signatures. These sites have been identified to be associated with a barrier against the zoonotic introduction of IAV into the human population. Previous experimental results indicated that adaptive mutations at these sites in the NP of the 1918 and 2009 pandemic strains could have contributed to increased resistance in murine Mx1 and human MxA [17].

**Table 3:** Count of host-specific genomic signatures in each internal protein.

Category\Protein	PB2	PB1	PA	NP	M1	M2	NS1	NS2	PB1-F2	Total
**Avian-Human**	20	14	15	20	5	14	18	4	19	129
**Swine-Human**	9	1	10	17	3	10	14	2	11	77

**Table 4:** Avian-human genomic signatures and their amino acid residues.

		Avian	Human			Avian	Human
Protein	Position^a^	AA (percent)^b^	AA (percent)^b^	Protein	Position^a^	AA (percent)^b^	AA (percent)^b^
**PB2**	271	T (97%)	A (99%)	**NP**	305	R (99%)	K (98%)
	588	A (95%)	IT (57% 42%)		33	V (99%)	I (98%)
	684	A (98%)	S (81%)		357	Q (99%)	K (98%)
	453	P (92%)	SH (44% 43%)		100	R (99%)	VI (73% 26%)
	292	I (86%)	TV (55% 40%)		313	F (98%)	YV (70% 28%)
	475	L (99%)	ML (57% 42%)		351	R (94%)	K (86%)
	559	T (91%)	IT (42% 28%)		136	L (84%)	IM (67% 32%)
	627	E (98%)	KE (56% 44%)		283	L (99%)	PL (70% 30%)
	368	R (98%)	KR (56% 43%)		61	I (98%)	LI (69% 30%)
	567	D (94%)	ND (55% 43%)		353	V (85%)	SI (50% 32%)
	613	V (97%)	TV (52% 44%)		16	G (96%)	DG (70% 30%)
	199	A (99%)	SA (57% 43%)		452	R (87%)	K (81%)
	674	A (94%)	TA (55% 44%)		214	R (97%)	KR (69% 31%)
	702	K (96%)	RK (56% 44%)		293	R (98%)	KR (68% 32%)
	64	M (98%)	TM (56% 43%)		422	R (100%)	KR (68% 32%)
	44	A (99%)	SA (56% 44%)		217	I (96%)	SV (48% 28%)
	105	T (97%)	VT (53% 43%)		442	T (99%)	AT (68% 32%)
	661	A (92%)	TA (55% 44%)		455	D (99%)	ED (68% 32%)
	590	G (84%)	SG (61% 38%)		372	E (98%)	DE (69% 31%)
	9	D (97%)	ND (53% 43%)		109	I (95%)	VI (66% 33%)
**PB1**	336	V (98%)	I (94%)	**M1**	115	V (98%)	IV (65% 34%)
	581	E (98%)	D (92%)		137	T (96%)	AT (65% 35%)
	361	S (96%)	RS (76% 21%)		121	T (93%)	AT (66% 34%)
	486	R (98%)	KR (76% 23%)		218	T (95%)	AT (55% 43%)
	741	A (97%)	S (78%)		227	A (90%)	TA (53% 46%)
	584	R (96%)	QR (77% 22%)	**M2**	14	G (92%)	E (97%)
	216	S (95%)	GS (73% 26%)		57	Y (99%)	HY (72% 26%)
	621	Q (95%)	RQ (73% 26%)		20	S (93%)	NS (74% 25%)
	430	R (84%)	K (79%)		54	R (98%)	LR (48% 24%)
	179	M (94%)	IM (55% 30%)		86	V (99%)	AV (73% 26%)
	298	L (97%)	IL (54% 45%)		11	T (89%)	IT (73% 26%)
	327	R (97%)	KR (54% 45%)		18	KR (58% 33%)	R (98%)
	517	I (98%)	VI (54% 46%)		78	Q (99%)	KQ (53% 26%)
	375	NS (56% 34%)	S (80%)		55	LF (62% 35%)	F (97%)
**PB1-F2**	76	V (99%)	A (89%)		16	E (85%)	GE (69% 31%)
	73	K (98%)	R (84%)		93	N (97%)	SN (75% 24%)
	87	E (93%)	G (91%)		28	IV (68% 27%)	VI (71% 26%)
	59	K (96%)	R (84%)		89	G (92%)	SG (53% 38%)
	79	R (92%)	Q (86%)		82	SN (77% 21%)	NS (66% 33%)
	60	Q (97%)	L (67%)	**NS1**	60	AE (55% 40%)	V (95%)
	31	E (91%)	GE (70% 28%)		114	SG (67% 24%)	P (98%)
	27	T (95%)	IT (66% 33%)		125	D (96%)	ED (77% 22%)
	82	L (92%)	SP (49% 30%)		48	S (95%)	NS (77% 23%)
	29	RK (67% 33%)	K (94%)		227	E (94%)	R (99%)
	62	L (95%)	PL (64% 35%)		70	EK (67% 29%)	K (98%)
	75	R (73%)	H (82%)		171	DT (63% 23%)	IY (41% 36%)
	6	D (90%)	GD (65% 35%)		81	I (95%)	MI (61% 38%)
	23	NS (66% 28%)	SD (73% 20%)		209	DN (66% 31%)	N (81%)
	25	Q (97%)	RQ (54% 45%)		59	RM (61% 24%)	HL (40% 37%)
	66	SN (61% 38%)	N (96%)		112	AT (57% 41%)	EI (41% 36%)
	42	CY (53% 47%)	Y (97%)		67	RD (71% 23%)	KW (40% 37%)
	16	IT (79% 20%)	TI (66% 34%)		215	P (79%)	TP (60% 38%)
	70	EG (55% 42%)	GV (49% 42%)		21	RL (74% 25%)	QR (60% 40%)
**PA**	356	K (98%)	R (98%)		22	FL (73% 26%)	VF (60% 38%)
	409	S (87%)	N (97%)		229	E (97%)	KE (66% 34%)
	382	E (92%)	D (83%)		18	VI (73% 26%)	IV (76% 23%)
	277	S (96%)	HY (39% 39%)		129	IT (65% 30%)	MV (36% 35%)
	204	R (99%)	KR (79% 20%)	**NS2**	70	S (89%)	G (99%)
	256	R (98%)	K (66%)		57	S (95%)	YL (39% 33%)
	268	L (99%)	IL (58% 41%)		89	IK (65% 20%)	TA (52% 36%)
	552	T (100%)	ST (59% 41%)		107	L (99%)	FL (58% 41%)
	337	A (95%)	SA (59% 40%)				
	225	S (99%)	CS (59% 40%)				
	55	D (99%)	ND (59% 40%)				
	28	P (100%)	LP (58% 41%)				
	404	A (94%)	SA (59% 41%)				
	57	R (97%)	QR (59% 40%)				
	100	V (95%)	AV (58% 41%)				

a Positions were sorted in the descending order of ARI.

b We showed only the dominant amino acid residues with more than 20% conservation.

**Table 5:** Swine-human genomic signatures and their amino acid residues.

		Swine	Human			Swine	Human
Protein	Position^a^	AA (percent)^b^	AA (percent)^b^	Protein	Position^a^	AA (percent)^b^	AA (percent)^b^
**PB2**	684	A (71%)	S (81%)	**NP(cont.)**	422	R (93%)	KR (68% 32%)
	292	IV (66% 23%)	TV (55% 40%)		214	R (91%)	KR (69% 31%)
	567	D (94%)	ND (55% 43%)		109	I (90%)	VI (66% 33%)
	453	PS (66% 29%)	SH (44% 43%)		375	D (85%)	GD (40% 29%)
	105	T (93%)	VT (53% 43%)		217	IV (70% 22%)	SV (48% 28%)
	44	A (93%)	SA (56% 44%)		353	IV (48% 42%)	SI (50% 32%)
	588	AT (45% 45%)	IT (57% 42%)		344	S (93%)	LS (51% 49%)
	674	A (90%)	TA (55% 44%)		34	G (86%)	DG (53% 46%)
	702	K (91%)	RK (56% 44%)		31	R (86%)	KR (55% 45%)
**PB1**	327	R (93%)	KR (54% 45%)	**M1**	115	V (92%)	IV (65% 34%)
**PB1-F2**	34	S (87%)	N (94%)		137	T (92%)	AT (65% 35%)
	71	Y (78%)	S (94%)		218	T (95%)	AT (55% 43%)
	29	R (80%)	K (94%)	**M2**	57	Y (93%)	HY (72% 26%)
	82	L (95%)	SP (49% 30%)		86	V (95%)	AV (73% 26%)
	23	N (66%)	SD (73% 20%)		93	N (96%)	SN (75% 24%)
	37	R (78%)	QR (73% 26%)		78	Q (96%)	KQ (53% 26%)
	62	L (83%)	PL (64% 35%)		54	R (85%)	LR (48% 24%)
	60	QP (56% 38%)	L (67%)		14	GE (56% 43%)	E (97%)
	83	SF (63% 34%)	F (95%)		89	G (95%)	SG (53% 38%)
	6	D (86%)	GD (65% 35%)		16	E (80%)	GE (69% 31%)
	27	T (80%)	IT (66% 33%)		82	S (85%)	NS (66% 33%)
**PA**	356	KR (62% 37%)	R (98%)		28	ID (42% 23%)	VI (71% 26%)
	204	R (80%)	KR (79% 20%)	**NS1**	114	SP (59% 39%)	P (98%)
	268	L (95%)	IL (58% 41%)		22	F (96%)	VF (60% 38%)
	552	T (96%)	ST (59% 41%)		81	I (94%)	MI (61% 38%)
	225	S (94%)	CS (59% 40%)		209	DN (64% 34%)	N (81%)
	277	SF (62% 21%)	HY (39% 39%)		206	R (49%)	SC (61% 37%)
	100	V (92%)	AV (58% 41%)		21	R (93%)	QR (60% 40%)
	337	A (85%)	SA (59% 40%)		211	R (96%)	GR (57% 42%)
	404	A (90%)	SA (59% 41%)		215	P (86%)	TP (60% 38%)
	28	PS (75% 20%)	LP (58% 41%)		95	L (95%)	IL (47% 38%)
**NP**	313	F (80%)	YV (70% 28%)		166	L (96%)	FL (54% 46%)
	283	L (93%)	PL (70% 30%)		171	DN (52% 29%)	IY (41% 36%)
	372	E (93%)	DE (69% 31%)		84	V (92%)	TV (41% 39%)
	293	R (94%)	KR (68% 32%)		227	G (76%)	R (99%)
	442	T (94%)	AT (68% 32%)		91	AT (55% 35%)	TS (62% 36%)
	455	D (94%)	ED (68% 32%)	**NS2**	107	L (96%)	FL (58% 41%)
	61	I (92%)	LI (69% 30%)		89	AI (33% 30%)	TA (52% 36%)
	16	G (92%)	DG (70% 30%)				

a Positions were sorted in the descending order of ARI.

b We showed only the dominant amino acid residues with more than 20% conservation.

**Table 6:** Comparison of avian-human genomic signatures.

Protein	New Signature^a^	Nonsignature^b^
PB2	453,559,590,684	81
PB1	179,216,298,327,361,375,430,486,517,581,584,621,741	–
PA	204,256,277	65,66,321,400,421
NP	109,217,293,351,353,372,422,442,452,455	375,423
M1	218,227	–
M2	16,18,82,89,93	–
NS1	18,21,48,59,67,70,112,114,125,129,171,209,229	84
NS2	57,89	60

a New genomic signatures identified in this study, but not reported in previous works [10], [12], [13], [16].

b Site previously reported to be a signature [10], [12], [13], [16], but not considered as a significant one in this study.

**Table 7:** Comparison of swine-human genomic signatures.

Protein	New Signature^a^
PB2	105,292,453,567,588,674,684,702
PB1	327
PA	28,100,204,225,277,337,356,404
NP	16,31,34,61,109,214,217,283,293,313,344,353,372,375,422,442,455
M1	115,218
M2	14,16,28,54,78,82,89
NS1	21,22,81,84,91,95,114,166,171,206,209,211,215,227
NS2	89

a New genomic signatures identified in this study, but not reported in previous works [10], [12], [16].

**Figure 2:**
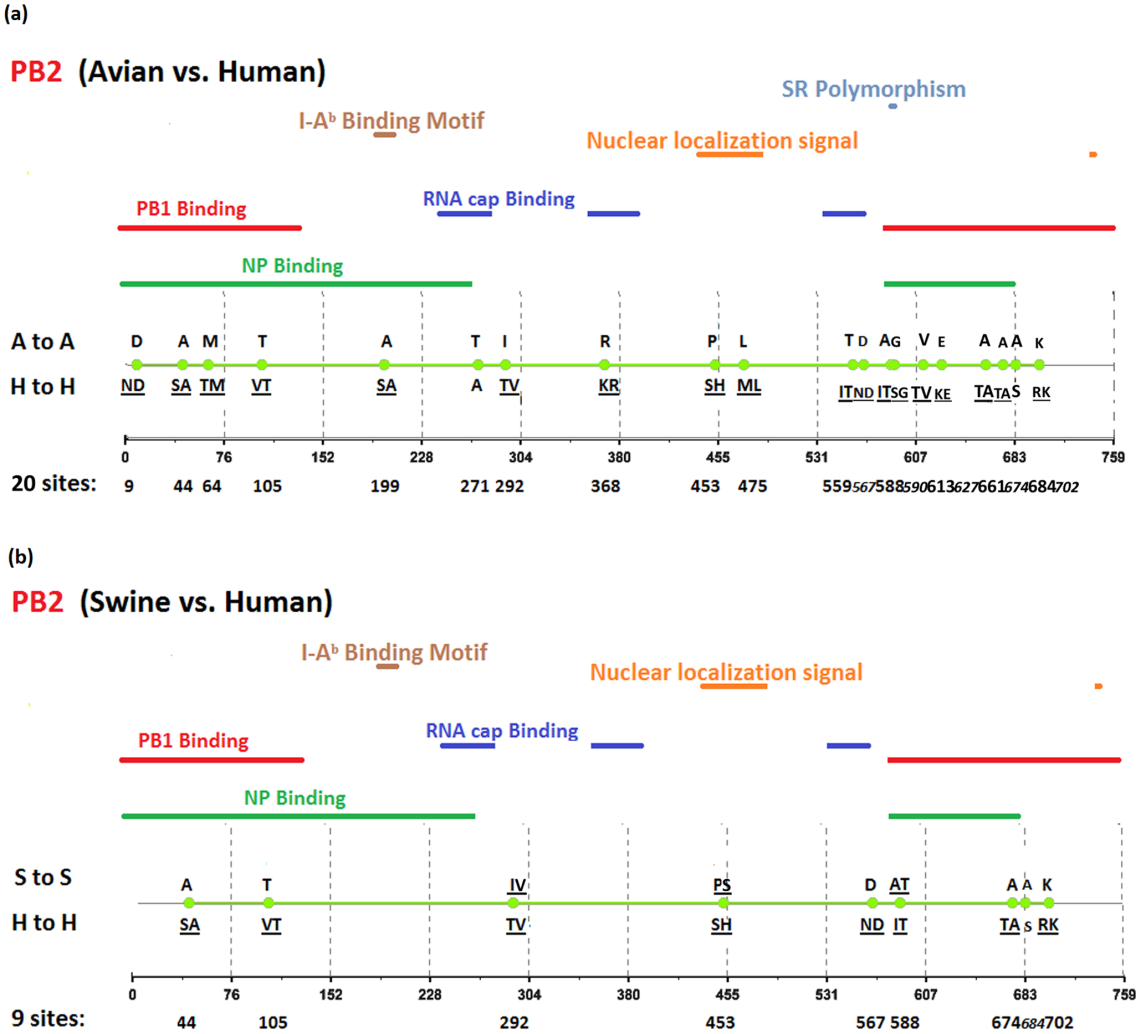
Genomic signatures in PB2: (a) avian vs. human (b) swine vs. human. Green circles denote the signatures in the PB2 sequence, with their positions shown on the bottom line. The dominant amino acid residues of the signatures are placed above (A to A or S to S) and under (H to H) the circles. The color lines indicate the functional or structural domains mapped by the signatures.

**Figure 3:**
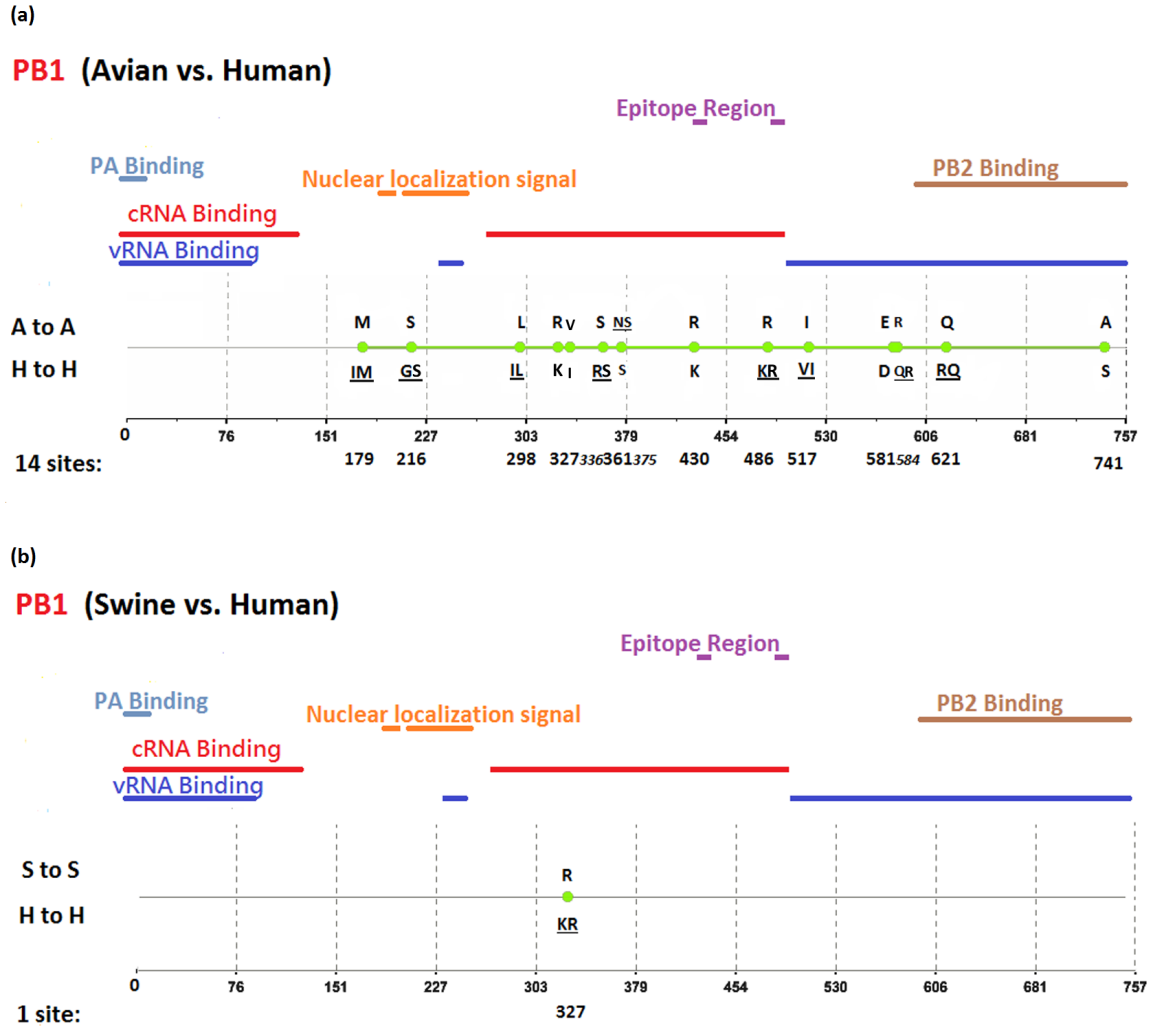
Genomic signatures in PB1: (a) avian vs. human (b) swine vs. human. Green circles denote the signatures in the PB1 sequence, with their positions shown on the bottom line. The dominant amino acid residues of the signatures are placed above (A to A or S to S) and under (H to H) the circles. The color lines indicate the functional, structural, or epitopic domains mapped by the signatures.

**Figure 4:**
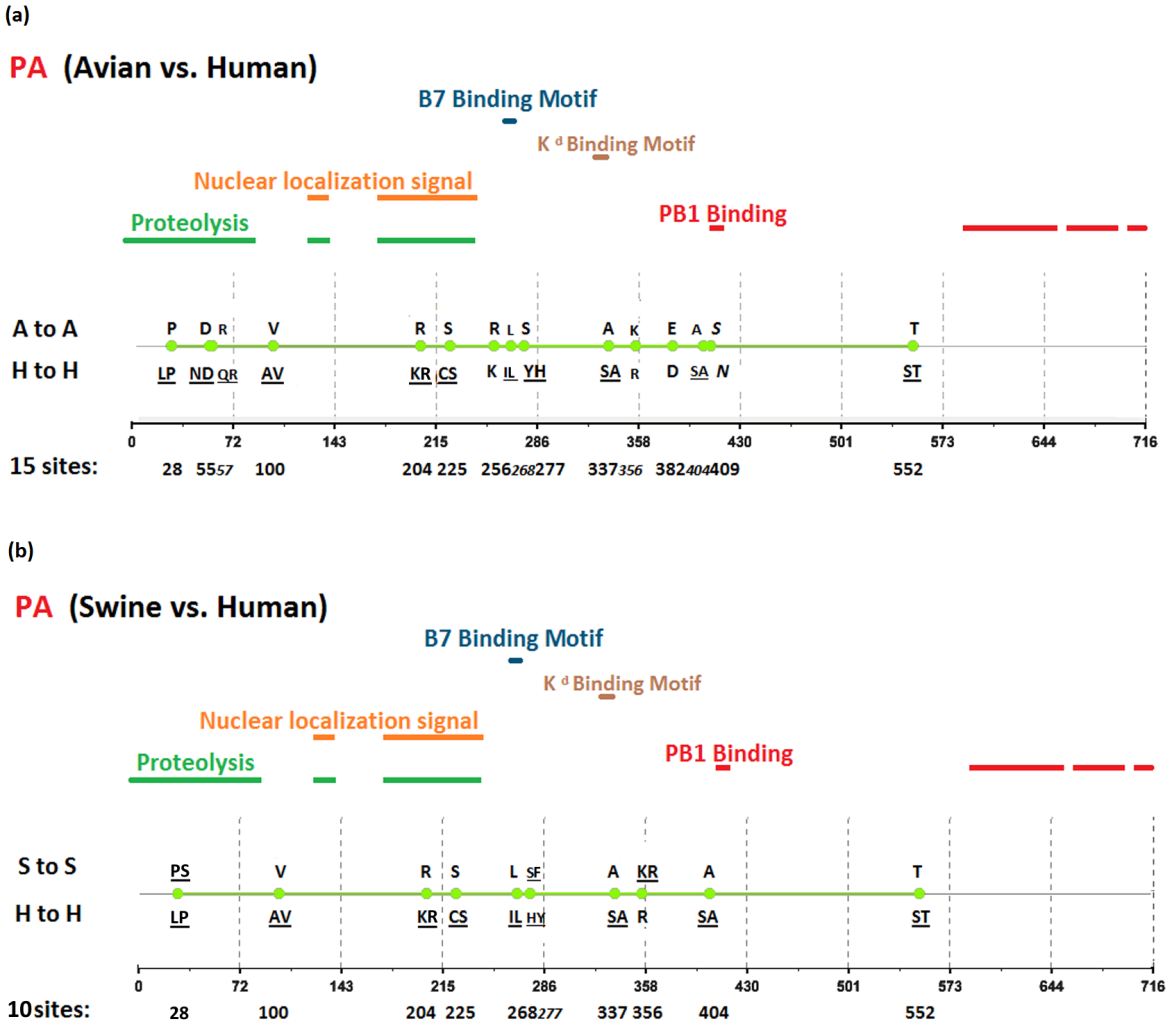
Genomic signatures in PA: (a) avian vs. human (b) swine vs. human. Green circles denote the signatures in the PA sequence, with their positions shown on the bottom line. The dominant amino acid residues of the signatures are placed above (A to A or S to S) and under (H to H) the circles. The color lines indicate the functional or structural domains mapped by the signatures.

**Figure 5:**
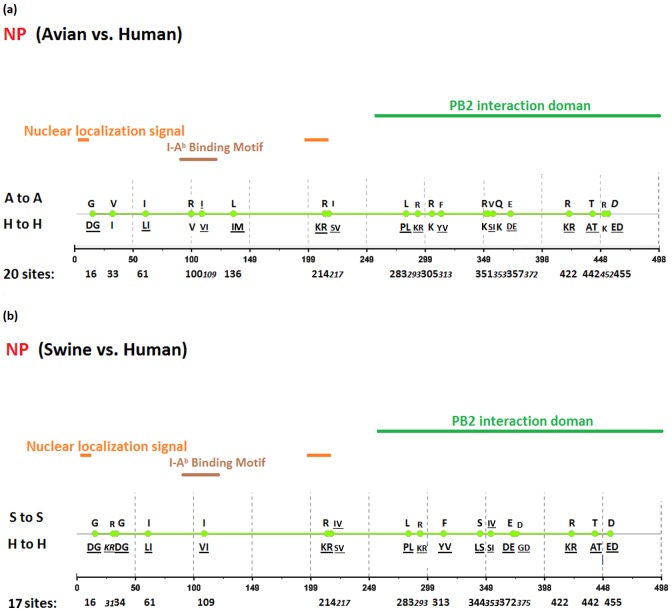
Genomic signatures in NP: (a) avian vs. human (b) swine vs. human. Green circles denote the signatures in the NP sequence, with their positions shown on the bottom line. The dominant amino acid residues of the signatures are placed above (A to A or S to S) and under (H to H) the circles. The color lines indicate the functional or structural domains mapped by the signatures.

**Figure 6:**
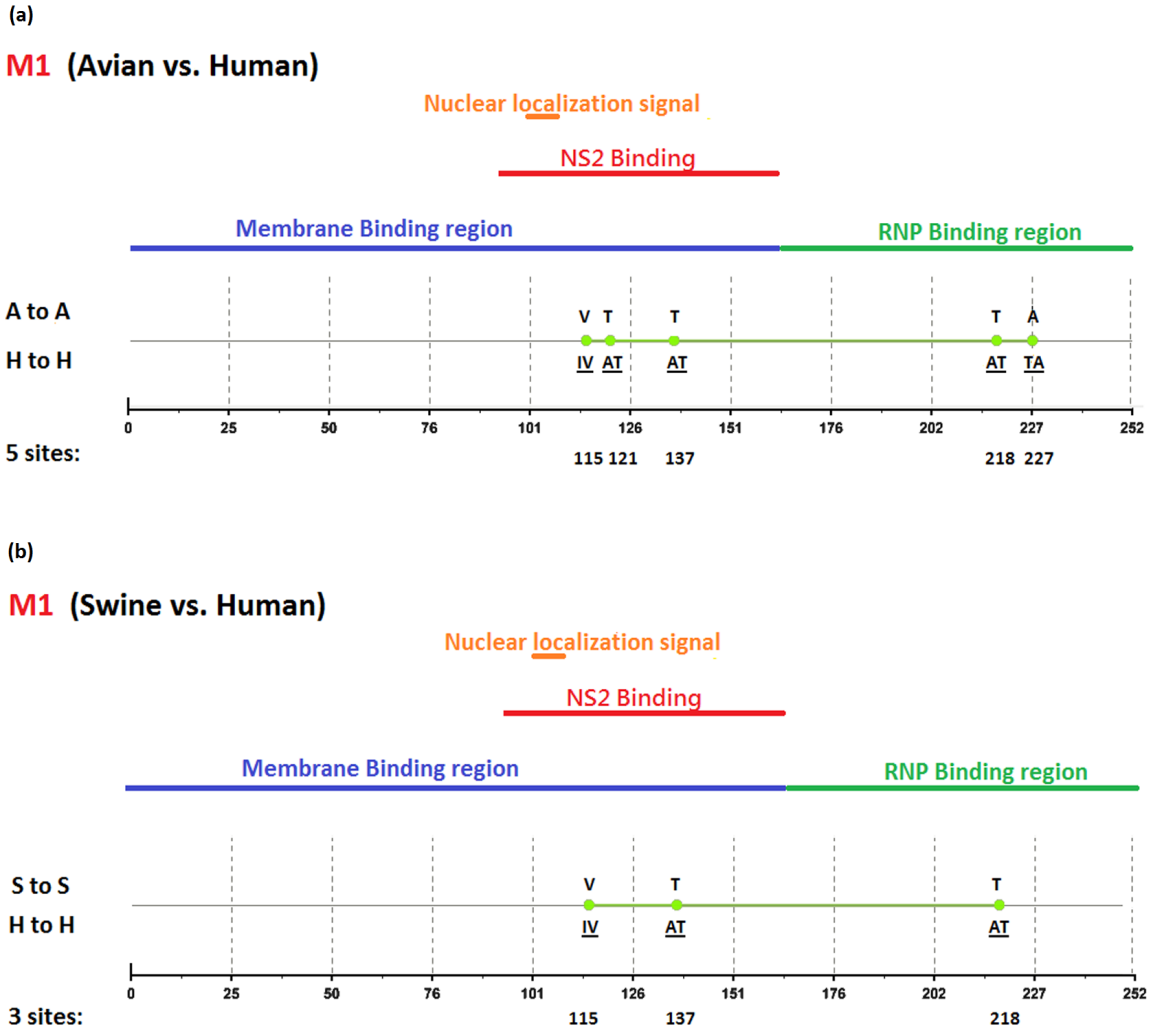
Genomic signatures in M1: (a) avian vs. human (b) swine vs. human. Green circles denote the signatures in the M1 sequence, with their positions shown on the bottom line. The dominant amino acid residues of the signatures are placed above (A to A or S to S) and under (H to H) the circles. The color lines indicate the functional or structural domains mapped by the signatures.

**Figure 7:**
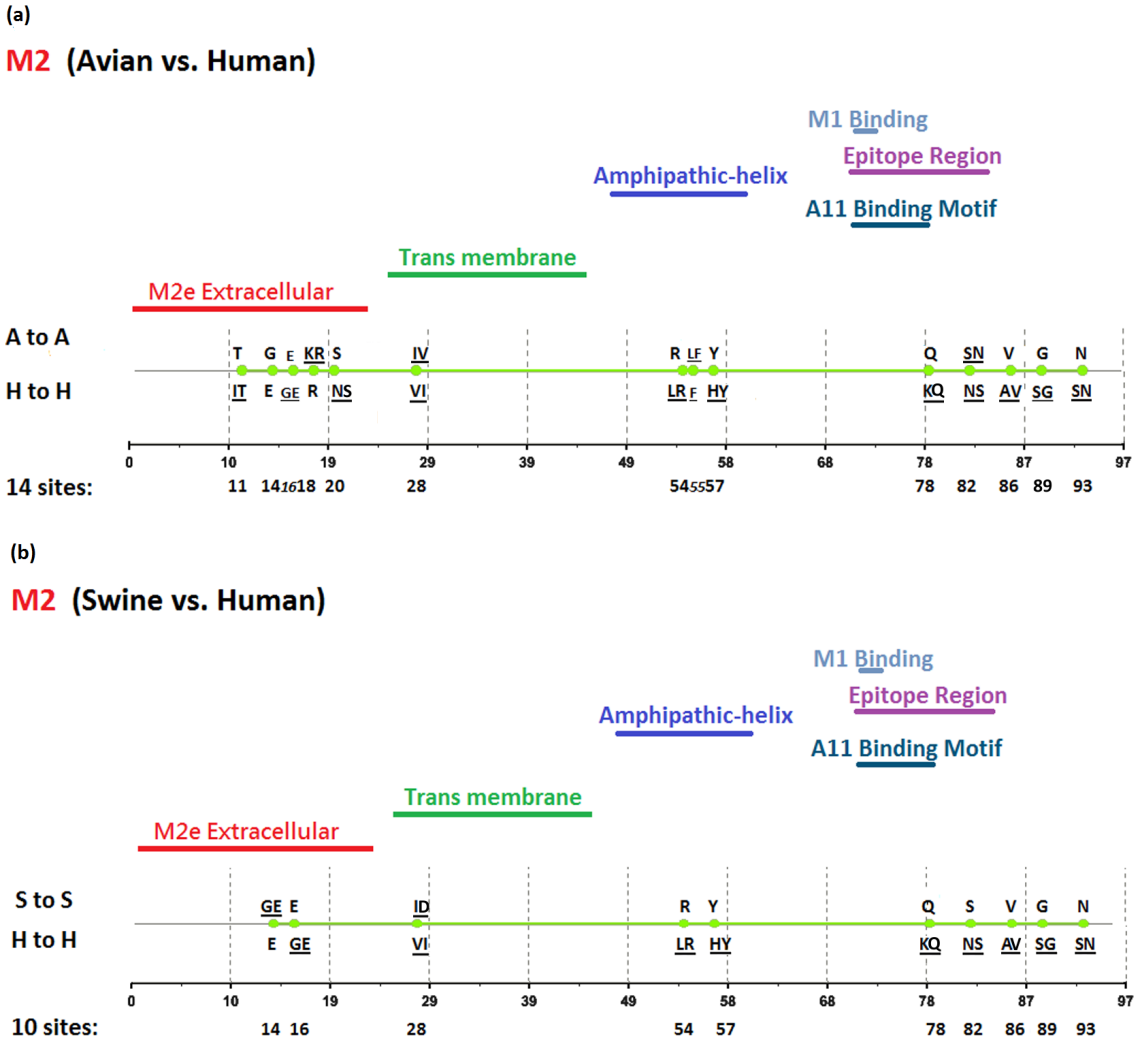
Genomic signatures in M2: (a) avian vs. human (b) swine vs. human. Green circles denote the signatures in the M2 sequence, with their positions shown on the bottom line. The dominant amino acid residues of the signatures are placed above (A to A or S to S) and under (H to H) the circles. The color lines indicate the functional, structural, or epitopic domains mapped by the signatures.

**Figure 8:**
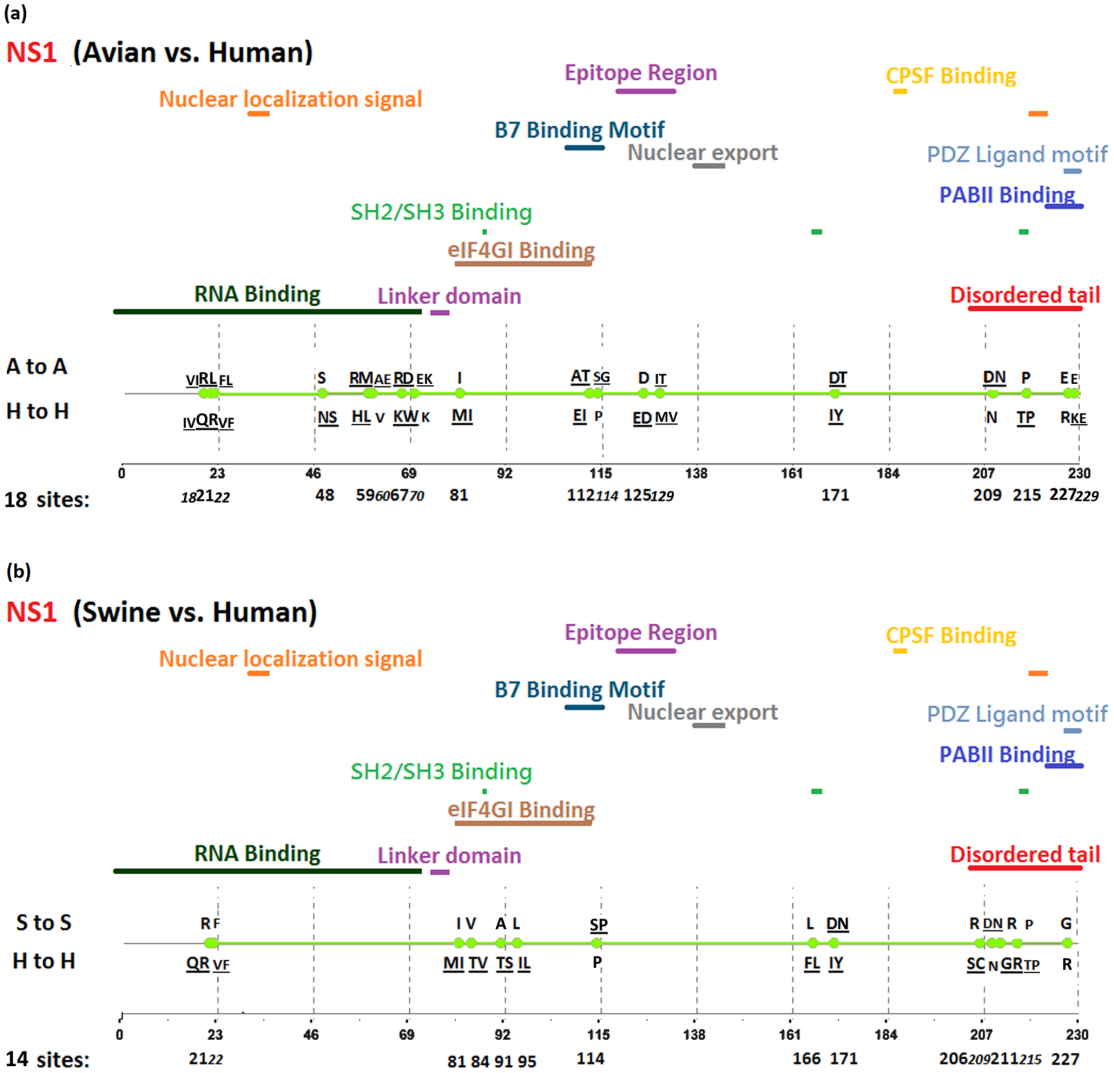
Genomic signatures in NS1: (a) avian vs. human (b) swine vs. human. Green circles denote the signatures in the NS1 sequence, with their positions shown on the bottom line. The dominant amino acid residues of the signatures are placed above (A to A or S to S) and under (H to H) the circles. The color lines indicate the functional, structural, or epitopic domains mapped by the signatures.

**Figure 9:**
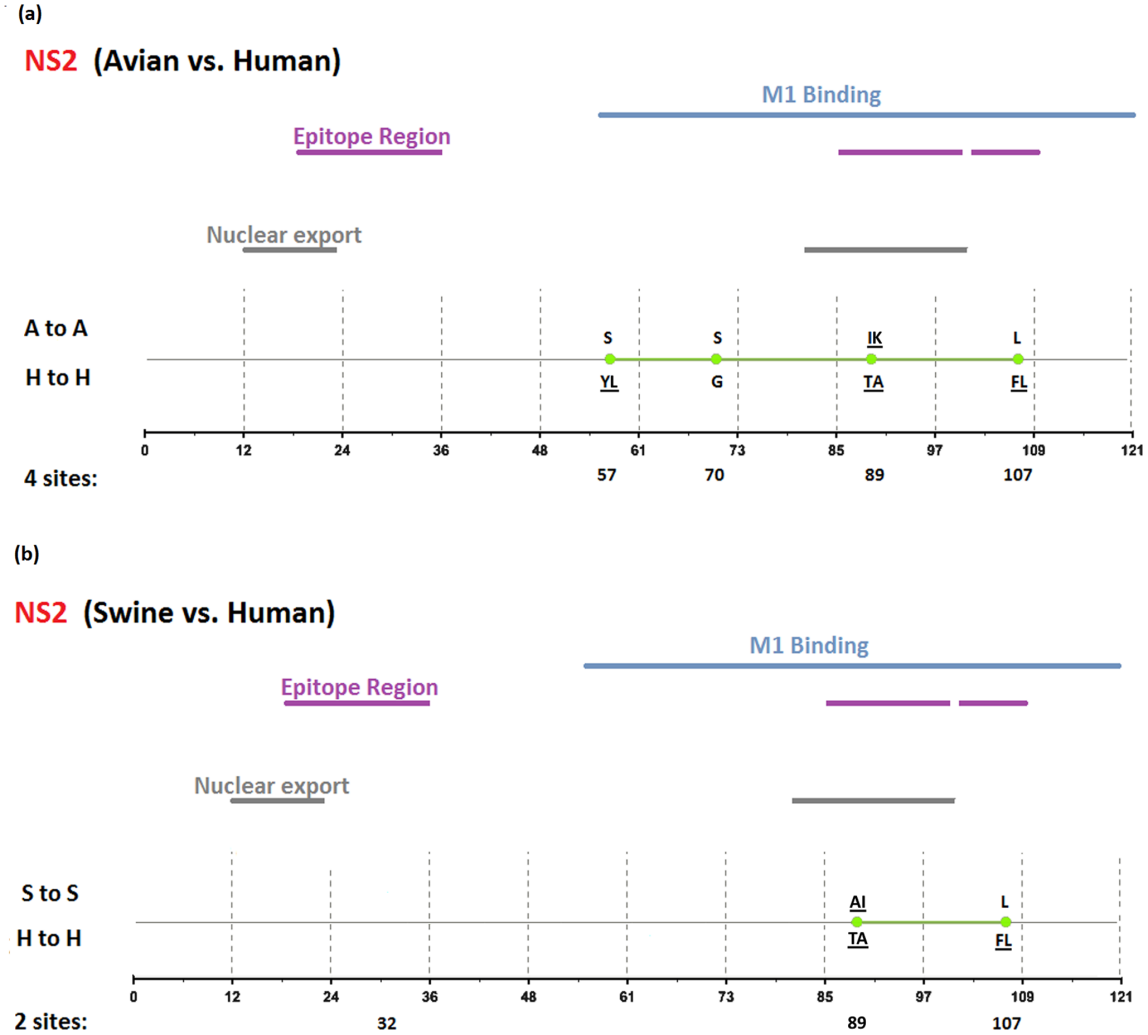
Genomic signatures in NS2: (a) avian vs. human (b) swine vs. human. Green circles denote the signatures in the NS2 sequence, with their positions shown on the bottom line. The dominant amino acid residues of the signatures are placed above (A to A or S to S) and under (H to H) the circles. The color lines indicate the functional, structural, or epitopic domains mapped by the signatures.

**Figure 10:**
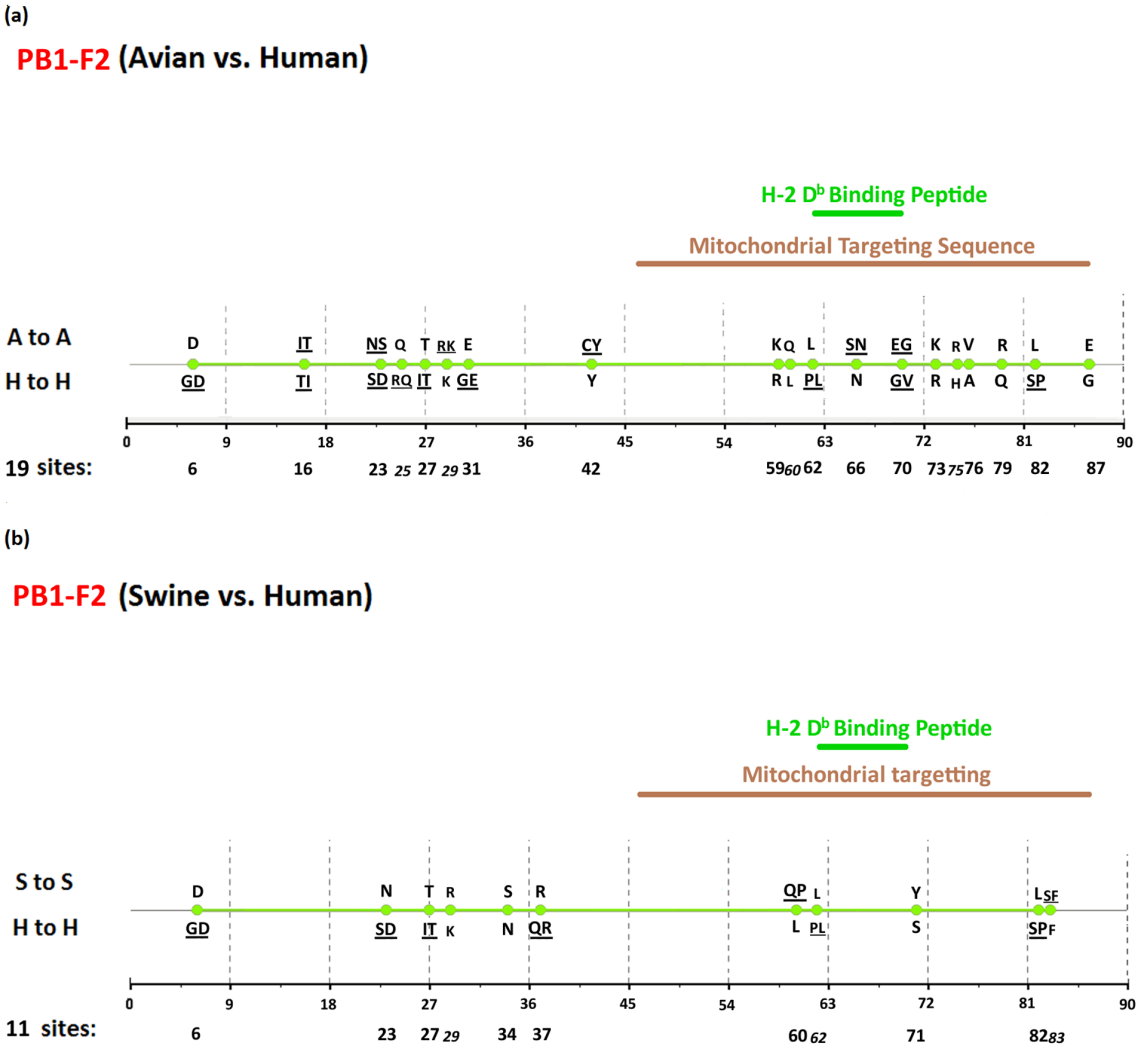
Genomic signatures in PB1-F2: (a) avian vs. human (b) swine vs. human. Green circles denote the signatures in the PB1-F2 sequence, with their positions shown on the bottom line. The dominant amino acid residues of the signatures are placed above (A to A or S to S) and under (H to H) the circles. The color lines indicate the functional or structural domains mapped by the signatures.

Figures 2, 3, 4, 5, 6, 7, 8, 9, and 10 show the positions of the genomic signatures in each of the 9 internal IAV proteins. These figures also show the functional, structural, or antigenic domains mapped by the signatures. Similar to the findings by Miotto et al. in avian sequences [12], we identified that the NP (20 sites), PB2 (20 sites), and PA (15 sites) were among the proteins that had the highest numbers of signatures. These proteins, together with the PB1 polymerase, form the RNP complex that encloses the genomic segments in the virion. Our analyses showed consistent results that most of the avian-human PB2 characteristic sites were located in the PB1 and NP binding areas. We observed similar results in the swine-human PB2 signatures (Figure 2). Unlike Miotto et al., who identified a single avian-human characteristic site (PB1–336) in PB1, we identified 14 sites, including PB1–336 (Figure 3). One notable signature we identified, which previous computational methods failed to recognize [5], [10], [12], [13], [16], was PB1–375. Its amino acid is an S in most human viruses and an N in most avian viruses. Previous studies of human pandemics showed a cross-species amino acid substitution at this site, and suggested an important role of PB1–375 in adaptation to mammals [6], [18].

The number of characteristic sites mapping to a reported domain was smaller in the PA protein than in the PB2 or PB1. Previous studies have identified some of these sites as located in proximity to the epitopic regions [19], [20], or in the proteolysis domain [21] and nuclear localization signal area [22], as shown in Figure 4. In the NP protein shown in Figure 5, most of the characteristic sites were involved in the PB2 interactions. A study by Mänz et al. verified that the mutations at 305, 351, 353, and 357 affect Mx1 resistance [17]. Our findings were in accordance with previous functional analyses implicating PB2 as a putative target of Mx1 [23]. Based on their positions in the sequence, the genomic signatures of M1 could be divided into 2 groups. As shown in Figure 6, the group in proximity to position 126 was within the membrane-binding region [24], and the other was located in the RNP-binding region [25]. The M2 protein contains 3 clusters of signatures and a single outlier M2–28 in the transmembrane region [26], as shown in Figure 7. The cluster of signatures to the left of the M2–28 is within the M2 extracellular region (M2e) [26], [27], the cluster of signatures to the right of the M2–28 represents part of an amphipathic helix [28], and the final cluster of signatures is located in the M2 protein tail, which reportedly interacts with the M1 protein [29]. Most of the signatures of NS1 were located in the RNA binding domain [30], [31], the eIF4GI subunit-binding domain [32], [33], or the epitopic regions [19], [34], as shown in Figure 8. Some of the signatures could be mapped to multiple domains, such as the NS1–215 and 227. By comparing the signatures identified in NS2 with the reported domains, we identified that all of the signatures constituted part of the M1 binding domain or the epitope (Figure 9). The genomic signatures of PB1-F2 could be divided into two parts by the position 42, as shown in Figure 10. The group of signatures to the right of PB1-F2-42 were mapped to the mitochondria targeting domain [35]. Among them we also identified PB1-F2-62, 66 and 70, which were part of the H-2 D^b^ binding peptide [36]. The other group of signatures were mostly clustered at positions 23–31. Further investigation is required to elucidate their their roles in polymerase activity. Table S1 in Materials S1 shows the reported domains in each internal protein.

### Identification of the Chronological Host-specific Genomic Signature

Genomic signatures can change over time because of point mutations or interspecies reassortments. According to the years in which previous human pandemics occurred, we divided the time between 1902 and 2013 into 6 periods: 1902–1918, 1919–1957, 1958–1968, 1969–1977, 1978–2009, and 2010–2013. We assigned each IAV to one of the 6 chronological groups according to its year of discovery. From each chronological group, we identified the chronological genomic signatures that were characteristic of the hosts and specific to that period. Table 8 shows the number of signatures in each period for the internal proteins.

**Table 8:** Numbers of host-specific chronological genomic signatures in 6 periods.

Category^a^	Protein	1902–1918^b^	1919–1957	1958–1968	1969–1977	1978–2009	2010–2013	mean(sd)
**A-H**	**PB2**	15	20	20	20	20	16	18.5(2.3)
	**PB1**	10	9	8	7	13	20	11.2(4.8)
	**PB1-F2**	N/A^c^	20	10	6	19	20	15(6.6)
	**PA**	17	20	20	20	20	15	18.7(2.2)
	**NP**	8	20	20	20	20	20	18.0(4.9)
	**M1**	4	6	4	5	5	6	5.0(0.9)
	**M2**	5	13	14	14	14	6	11(4.3)
	**NS1**	11	10	6	20	19	20	14.3(6.1)
	**NS2**	2	5	5	6	5	11	5.7(2.9)
**S-H**	**PB2**	N/A	20	20	20	4	8	14.4(7.8)
	**PB1**	N/A	18	20	20	3	14	15.0 (7.1)
	**PB1-F2**	N/A	N/A^c^	N/A^c^	11	15	18	14.7(3.5)
	**PA**	N/A	20	20	20	8	4	14.4(7.8)
	**NP**	N/A	20	20	20	15	2	15.4(7.8)
	**M1**	N/A	4	5	5	3	0	3.4 (2.1)
	**M2**	N/A	11	12	11	10	1	9(4.5)
	**NS1**	N/A	20	20	20	20	12	18.4(3.6)
	**NS2**	N/A	8	12	10	2	0	6.4(5.2)

a A-H: Avian-Human; S-H: Swine-Human.

b The period 1902–1918 was excluded from the study of swine-human signatures, denoted by N/A, because of the lack of swine data during 1902–1918.

c The period 1902–1918 for PB1-F2 was excluded from the study of avian-human signatures, denoted by N/A, and so were the periods 1919–1957 and 1958–1968 for swine-human because of the lack of data.

As shown in Table 8, the PB2, PA, and NP proteins had the largest average numbers of avian-human chronological genomic signatures. These findings were consistent with the results from genomic signature analyses during 1902–2013, as shown in Table 3. When examining the numbers of signatures across all periods, we observed that the numbers of chronological signatures in the PB2, PA, and NP proteins were relatively stable, except during 1902–1918. A stable number of signatures over time suggested that the PB2, PA, and NP share similar evolutionary pathways, and a large number of characteristic sites indicated that they undergo rigorous multigenic adaptation to a new host. These findings supported the hypothesis that the coevolution of RNP proteins is a crucial factor that restrains the genomic segments from forming interspecies reassortants, and limits the evolutionary divergence between host-specific lineages [2]. As shown in Table 8, we observed that the average swine-human chronological genomic signatures in the RNP proteins were among the largest, excluding NS1. Unlike the avian-human chronological signatures, the numbers markedly reduced during 1978–2009 and 2010–2013. These findings suggested that the sequence-level genetic differences in the RNP proteins between swine and human viruses might have reduced in recent years. We further observed that the number of PB1 chronological signatures reduced from 20 to 3 during 1969–1977 and 1978–2009, but increased to 14 during 2010–2013. These observations indicated the association of the viral polymerase complex with the swine-origin influenza virus (S-OIV) that caused the 2009 H1N1 pandemic, and showed the genetic diversity and adaptation variability in the components of the RNP complex in different hosts.

Aside from the RNP complex, the NS1 protein displayed maximum variance in the numbers of chronological avian-human signatures. The high variance in the number of the NS1 signatures resulted from selected signatures in one period becoming nonsignatures in a different period, or vice versa (see details of the avian-human NS1 signatures in each of the 6 periods in Table S2 in Materials S1, and Figure S1). The numbers of signatures markedly increased after 1968. Several signatures displayed an increase in ARI (ΔARI >0.2) from 1958–1968 to 1969–1977, and 5 signatures showed a peak ARI value during 1969–1977: NS1–23, 56, 98, 112, and 119 (Figure S1). Previous studies on A/HongKong/1/1968(HK-wt) showed NS1–23 and 98 to play crucial roles in adaptation and virulence in a novel host [37]–[39]. Our findings of peak ARI values for NS-23 and 98 during 1969–1977, immediately after 1968, corresponded well with those results. Our analyses of the ARI values of chronological genomic signatures could potentially elucidate the relationships between the chronological properties of signatures and the viral fitness of influenza viruses.

Our study’s analyses further showed that the number of M1 avian-human signatures remained relatively constant: between 4 and 6 throughout all periods. Although the numbers of signatures remained relatively stable during each period, the signatures identified in each period varied in their positions and amino acid residues (see details of the avian-human chronological genomic signatures of M1 in Table S3 in Materials S1, and Figure S2). When comparing the M1 signatures identified during 1902–1918 (M1–101, 121, 144, and 234) with those identified during 1958–1968 (M1–115, 121, 137, and 218), we observed that 4 signatures were identified during both periods, but their sites differed, with the exception of M1–121. We observed a marked reduction in the ARI values of M1–101, 144, and 234 from 1902–1918 to 1958–1968 (Figure S2). In contrast, the ARI values of M1–115, 137, and 218 increased substantially from 1902–1918 to 1958–1968. These results showed that the host-associated genomic signatures can change over time, and that the degree of change can vary in distinct sites in different proteins, as reflected in the various changes in the ARI values.

### Analysis of Host-specific Genomic Signature Transitions

To further investigate the chronological genomic signatures, we analyzed the transitions of the amino acid residues of the signatures in each internal protein across different periods. In the validity analyses, we examined the changing roles of the characteristic sites (signature or nonsignature) during different periods. These results provided information on the relationships between amino acid substitutions and host range phenotypes, and could increase our understanding of the effects of genetic diversity on the adaptation of the IAV. In the identity analyses, we examined the characteristic sites that remained valid throughout all periods for changes in their amino acid residues over time. The details of the transitions of the amino acid residues on the characteristic sites in each internal protein during the 6 periods are given in Tables S4 and S5 (in Materials S1). The various amino acid transitions in the characteristic sites might indicate the differences in the pathogenic and adaptive mechanisms of the IAV during different periods.

We identified a distinct amino acid transition pattern in PB2–590 and 591. Both signatures became valid after 2009, following a G590S and a Q591R mutation, respectively (Table S4 in Materials S1). The amino acid transitions in PB2–627 showed a contrasting pattern: PB2–627 maintained a valid signature from 1902 to early 2009, but after 2009 the dominant amino acid of PB2–627 in the human strain changed from K to E, as in the avian strain. Previous studies have conducted biochemical and modeling experiments to investigate the adaptive strategies of influenza viruses to evade restriction in hosts [40]–[43]. Our results on signature transitions supported the findings by Mehle et al. on the SR polymorphism that enables glutamic acid at position 627 to evade restriction in human cells [41]. These observations suggested that signature transition analysis can be applied as a preprocess to identify prospective functional sites in influenza viral proteins prior to further biochemical investigations. We also observed that the PB2–54, 65, 147, 184, 225, 315, 340, 559, and 645 showed similar transition patterns to those of the PB2–590 and 591 (Table S4 in Materials S1). Most of these sites are located in the NP binding domains (Table S1 in Materials S1, and Figure 2). In addition to the PB2–627, which switched from a signature to a nonsignature during 2010–2013, the PB2–199, 475, and 567 showed a similar signature-to-nonsignature tendency. These sites are involved in NP binding, nuclear localization signaling, and RNA cap binding. Further investigation is required to elucidate their roles in polymerase activity.

Most of the characteristic sites served as genomic signatures (i.e., valid sites) within specific periods. For example, M2–18 became a signature after 1958, and NS1–74 became a signature after 2010. Very few characteristic sites remained valid throughout all periods. The dominant amino acid residues on a characteristic site can vary across different periods or remain the same. For example, the dominant amino acids of NS1–67 in the avian and human IAV varied throughout different periods, whereas the dominant amino acids of M2–18 remained the same in the human virus and varied in the avian virus. In the PB2 protein, we identified one characteristic site, PB2–271, as a valid signature specific to avian and human IAV across all periods. Its amino acid transitions were a mutation from A to T in avian IAV and from T to A in human IAV during 1902–1918, as shown in Table S4 in Materials S1. In the signature analyses comparing swine and human IAV, the dominant amino acids of the PB2–271 in the swine virus switched from T to A during 1978–2009, and 2010–2013, as shown in Table S5 in Materials S1. A study by Kendra et al. showed that the mutation T271A in PB2 increases polymerase activity and virus growth in human cells. Results from in vitro reporter gene and sequence analyses indicated that the PB2-271A in S-OIV was likely to have contributed to its efficient transmission among humans during the 2009 H1N1 pandemic [44]. Our findings on the PB2-T271A in the swine virus further supported this hypothesis. Although a previous study using phylogenetic modeling [13] excluded the PB2–271 as a characteristic site, our analysis of the chronological genomic signature transitions successfully identified its relevance in host adaptation. In addition to the 271A mutation, the authors identified another PB2 mutation A588I that increased polymerase activity in mammalian cells [44]. Our results support the occurrence of an adaptive mutation of the conserved residues from A to I at site 588 in the human virus (Table S4 in Materials S1). Although PB2–588 had a conserved amino acid A in the avian virus throughout all periods, it showed a transition from A to T in the swine virus during 1978–2009 and 2010–2013. However, in the human virus, the PB2–588 showed an early transition from A to I, which later changed to T, as in the swine virus (Tables S4, S5 in Materials S1). Further investigation is required to establish if the change from I to T in the human virus reduced polymerase activity in human cells and contributed to the end of 2009 H1N1 pandemic. In addition to the PB2–271, the NP-100, 136, and 313 remained genomic signatures throughout all periods (Table S4 in Materials S1). Like PB2–271, their dominant amino acids showed chronological changes, but only in the human virus. Our identity analyses showed that only NP-33, 357, and M2–14 remained signatures throughout all periods and also maintained the same dominant amino acids (Table S4 in Materials S1). The stabilities of these sites indicate a crucial association with host range phenotypes and pathogenic mechanisms, which requires further verification.

## Discussion

The availability of a considerable amount of data on the IAV has enabled computational approaches to identify amino acid residues as host-specific genomic signatures. Previous studies have performed large-scale complete-proteome analyses of the IAV sequences [10], [12], [16]. Our study used more recent sequence data from the NCBI database (in February, 2013) than those studies. Unlike earlier computational methods that relied on a threshold to discriminate signatures from nonsignatures, such as the MI threshold of 0.4 in a study Miotto et al. [12], and the entropy threshold of 0.33 in a study by Chen and Shih [10], we used the ARI to evaluate and compare the ability of each site in the IAV sequences for the distinguishing of a host. Tamuri et al. speculated that the verification of characteristic sites in different viral proteins based on one single threshold is questionable because different viral proteins evolve according to different selective constraints [13]. The appropriate threshold might differ in different viral proteins because of their distinct characteristics and changes they might undergo during various circumstances. In addition, a threshold requires adjustment after novel data becomes available. For example, Chen and Shih changed their entropy threshold from 0.4 to 0.33 after an increase in the number of the identified IAV protein sequences [10].

For comparison, we calculated the entropy and the MI for each site. Low entropy in a site indicates that its amino acid residues are well-conserved, and thus the site is likely to represent a candidate genomic signature [10]. However, an approach that identifies signatures based on low entropy can overlook potential characteristic sites. For example, in the sequences in the NCBI from 1902 to 2013, the amino acid residues at position PB2–588 are dominated by A (95%) in avian viral sequences. However, in human viral sequences, the identical position is relatively equally dominated by I (57%) and T (42%). Although Chen and Shih considered PB2–588 in the sequences as of May 28, 2009 in the NCBI to be a genomic signature [10], the entropy-based method could mistakenly eliminate PB2–588 as a characteristic site from more recent sequence data because of the high entropy of PB2–588 in the human strain. We could have included PB2–588 as a signature by ignoring the entropy constraint, but this would have incurred an increase in the false-positive rate. In contrast, PB2–588 could be considered a signature because of its high MI and AR values. Because MI and ARI are different measures, instead of comparing their values to determine which measure is more effective in signature evaluation, we compared the rankings of the sites, and their conserved amino acid residues, according to MI and ARI. Table S6 in Materials S1 shows the top 20 MI- and ARI-ranked sites in avian and human PB2 proteins. PB2–645 and 591, the 19th- and 20th-ranked sites according to MI, have the same dominant amino acid in avian and human proteins. Though PB2–81, the 12th-ranked site, has different dominant residues between avian and human (T vs. M), the conservation levels of T and M in human are almost the same (M = 42.98% vs. T = 42.77%). Therefore, none of these sites is an appropriate genomic signature. In contrast, all the top 20 ARI-ranked sites showed differences in the dominant amino acid residues. We observed similar trends in other internal proteins. Overall, these findings suggested that the ARI provides a more appropriate measure for the ranking of characteristic sites when compared with MI.

Our experimental results showed that the ARI provides a more effective measure for detecting host-associated characteristic sites than entropy or MI. Using the IAV data in the NCBI, we identified novel signatures in 9 internal viral proteins that previous approaches failed to recognize. Several of the signatures could be mapped to known structural, functional, or antigenic domains of the proteins, which suggested their molecular functions and indicated the value of our approach.

Point mutations or interspecies reassortments can change the genomic signatures in viral sequences. Some previous studies analyzed adaptation trends in the previously identified genomic signatures without considering their phylogenetic relationships [10], [12], [16]. Other studies considered the phylogenetic structures, but applied theoretical modeling of site substitution rates [13]. In addition to providing updated data on the host-specific genomic signatures (Tables 4 and 5), our study analyzed the genomic signatures in their chronological order. We initially grouped the protein data chronologically, identified the signatures from each separate group, and then analyzed their variations. Therefore, unlike previous studies’ methods, our approach included the identification and analysis of sequence signatures and their chronological relationships. For example, from the chronological analysis of avian-human signatures, we detected a difference in the transition pattern in PB1 when compared with the other internal proteins: a comparatively larger number of characteristic sites had the same (or similar) dominant amino acid residues in the avian and the human viruses during 1919–1957 and 1958–1968, and the dominant residues varied after 1968 mostly in the human virus, but rarely in avian (Table S4 in Materials S1). These observations supported the reassortment hypothesis that PB1 gene was introduced from avian to human prior to the 1957 pandemic, and was maintained in human until 1968 [18], [45]. In addition, the chronological analysis of swine-human signature transitions during 1978–2009 further showed that there were relatively more characteristic sites with the same dominant residues in PB1 than in the other internal proteins (Table S5 in Materials S1), and the number of signatures dropped from 20 to 3, the minimum across all periods (Table 8). These observations indicated that the genetic variability in PB1 between swine and human was minimal during 1978–2009. Several studies into the lineages and evolutionary genomics of the 2009 S-OIV suggested that the PB1 of S-OIV emerged from a triple-reassortment virus circulating in North American swine, and the PB1 gene in the source triple-reassortant was derived from human at the time of the triple reassortment events in 1998 [45]–[47]. Our findings of the chronological signature transition patterns in PB1 were in accordance with the reassortment history of the S-OIV. Further investigation is required to identify other chronological transition patterns of the other internal proteins, and to verify their relations to the historical reassortment events.

Chronological genomic signatures provide the basis for novel types of investigation into the multiple genetic determinants of a host range. The numbers of chronological signatures during different periods and their variance can correlate with the level of rigorousness of multigenic adaptation of influenza viruses to a new host. A larger number of signatures indicates greater difficulty in the transmission and adaptation of a viral protein to a new species, whereas larger variance in the number of signatures across different periods suggests a wide variety of amino acid residues switching from signature to a nonsignature roles or vice versa. For example, the larger number of chronological genomic signatures in the PB2, PB1, PA, and NP proteins (Table 8) explains the occasional, but rare, transmission or adaptation of avian influenza viruses to humans [2]. The fluctuations in the ARI value for a signature across different periods indicate its genetic variability, through mutations or reassortment events, with time. Signatures with similar patterns in their ARI values are likely to be involved in related molecular functions and activities. Our analyses suggested correspondence between the ARI patterns (NS1–23 and 98) and increased viral growth [39].

The changes in the conserved amino acid residues of chronological genomic signatures throughout different periods indicate a chronological relationship between signature transitions and adaptation trends. Based on the chronological transitions of a signature, we can evaluate its stability according to validity and identity. Our results showed a unique transition pattern in the dominant amino acid residues of PB2–590 and 591, which was consistent with previous biochemical modeling results on the SR polymorphism [41]. We also identified other chronological signatures with similar amino acid transitions, such as the PB2–54, 65, and 147. According to the domains to which the chronological signatures are mapped, we were able to identify the variations in the domains during different periods. The chronological associations between the signatures and the mapped domains could provide alternative insight into previous findings on influenza virus evolution. Our analytical approach could serve as a preprocess to identify prospective characteristic sites that warrant further investigation.

## Conclusion

In this study, we proposed an alternative measure, based on the ARI, for the evaluation of the abilities of the genomic signatures of the IAV to distinguish the host. Using the data in the NCBI (in February, 2013), we identified 129 avian-human and 77 swine-human genomic signatures, including novel signatures that previous methods failed to recognize. Several of these novel signatures could be mapped to known domains to show the biological significance of the novel signatures, and indicate the value of the ARI in the evaluations. These novel signatures could potentially increase our understanding of genetic determinants and their potential combinations involved in host restriction. To chronologically analyze the genomic signatures, we divided the virus data into chronological groups, and then identified the genomic signatures from these groups. A comprehensive analysis of the chronological signatures throughout different periods indicated adaptation trends that were consistent with previously published results. Our chronological approach considers the underlying phylogenetic relationships of genomic signatures, and can identify adaptation trends more accurately than existing approaches that do not consider the evolutionary correlations among viral proteins.

## Supporting Information

Figure S1 **The ARI of NS1 chronological signatures in each period.** The X-axis shows the periods; the Y-axis represents the ARI. Several signatures show similar ARI transition patterns over the periods, such as NS1–23, 56, 98, 112, and 119.
(TIF)

Figure S2 **The ARI of M1 chronological signatures in each period.** The X-axis shows the periods; the Y-axis indicates the ARI. Several signatures show a marked increase in ARI during 1978–2009 and 2010–2013: M1–30, 116, 142, 207, 209, and 214. M1–30, 116 and 142 are located in the membrane binding region; 207, 209 and 214, in the RNP binding region.
(TIF)

Materials S1 **Supporting information of host-specific genomic signatures and transitions of characteristic sites.** Table S1: Catalogue of reported domains in 8 internal proteins. Table S2: NS1’s chronological genomic signatures identified in 6 periods and their amino acid residues. Table S3: M1’s chronological genomic signatures identified in 6 periods and their amino acid residues. Table S4: Transitions of amino acid residues on avian-human characteristic sites. Table S5: Transitions of amino acid residues on swine-human characteristic sites. Table S6: Top 20 sites in PB2 and their amino acid residues.
(DOC)
