# Correction: Identification and Chronological Analysis of Genomic Signatures in Influenza A Viruses

**DOI:** 10.1371/journal.pone.0095902

**Published:** 2014-04-16

**Authors:** The PLOS ONE Staff

**Notice of Republication**

This article was republished on March 31, 2014, due to the release of confidential or copyrighted information. The publisher apologizes for this error. Please download the article again to view the corrected version.
