# Supplementary figures and images for: Identification and Chronological Analysis of Genomic Signatures in Influenza A Viruses
Source: PLoS One. 2014 Jan 8;9(1):e84638. doi: 10.1371/journal.pone.0084638 (PMC3885579; doi:10.1371/journal.pone.0084638)

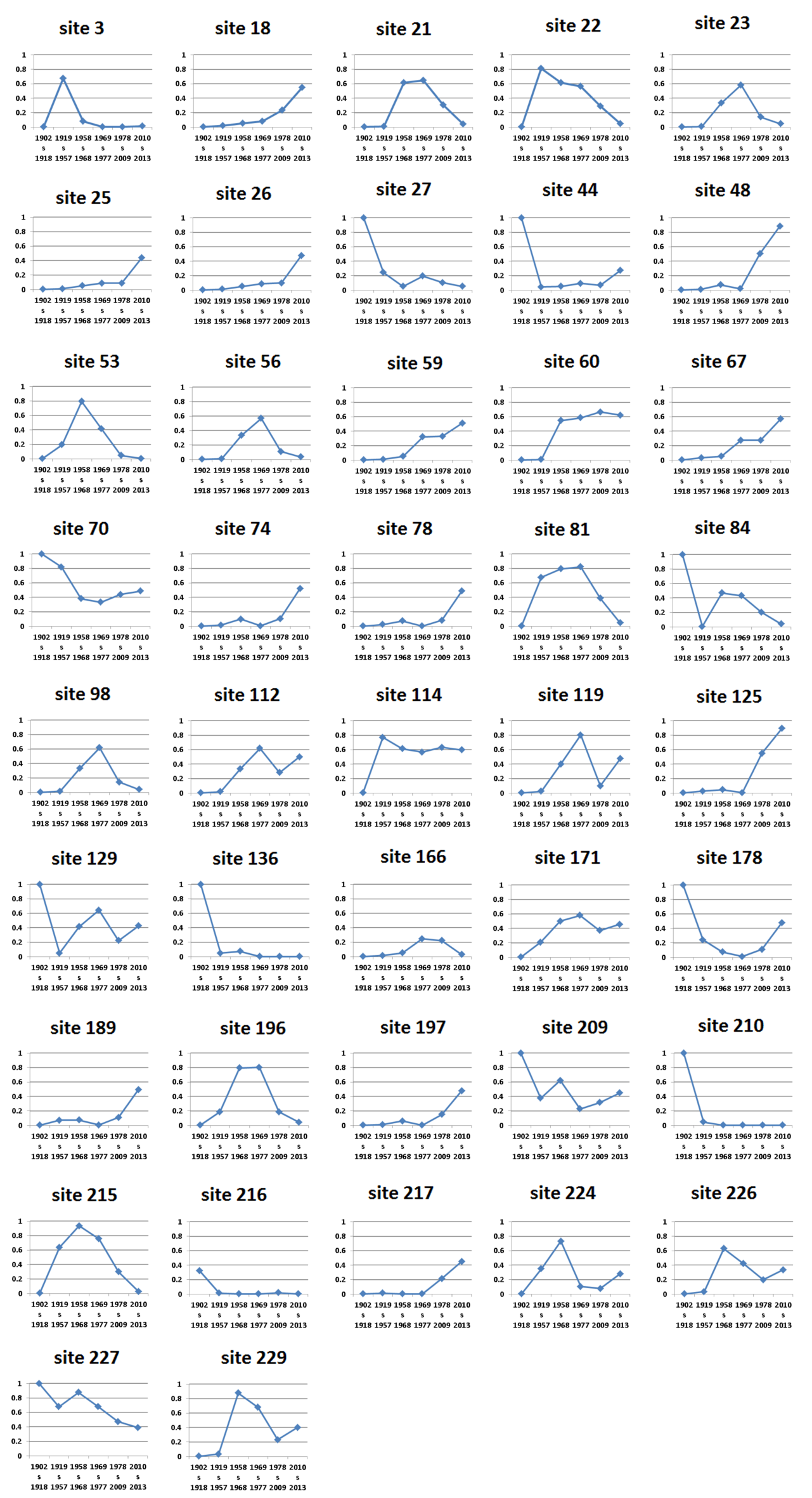

Supplement: Figure S1 — The ARI of NS1 chronological signatures in each period. The X-axis shows the periods; the Y-axis represents the ARI. Several signatures show similar ARI transition patterns over the periods, such as NS1–23, 56, 98, 112, and 119. (TIF) [file pone.0084638.s001.tif]

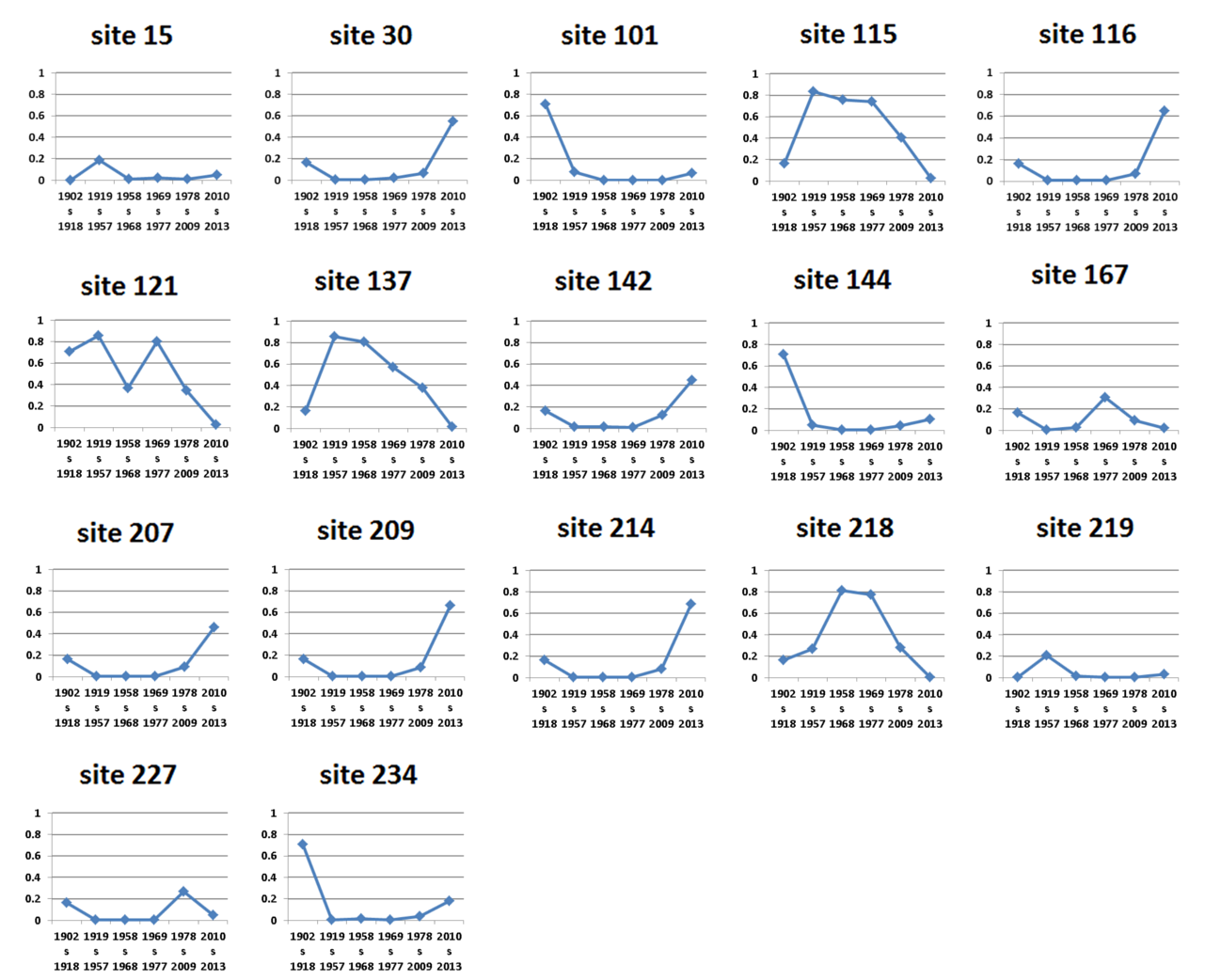

Supplement: Figure S2 — The ARI of M1 chronological signatures in each period. The X-axis shows the periods; the Y-axis indicates the ARI. Several signatures show a marked increase in ARI during 1978–2009 and 2010–2013: M1–30, 116, 142, 207, 209, and 214. M1–30, 116 and 142 are located in the membrane binding region; 207, 209 and 214, in the RNP binding region. (TIF) [file pone.0084638.s002.tif]
