# Supplementary material for: Identification and Chronological Analysis of Genomic Signatures in Influenza A Viruses
Source: PLoS One. 2014 Jan 8;9(1):e84638. doi: 10.1371/journal.pone.0084638 (PMC3885579; doi:10.1371/journal.pone.0084638)
Supplement: Materials S1 — Supporting information of host-specific genomic signatures and transitions of characteristic sites. Table S1: Catalogue of reported domains in 8 internal proteins. Table S2: NS1’s chronological genomic signatures identified in 6 periods and their amino acid residues. Table S3: M1’s chronological genomic signatures identified in 6 periods and their amino acid residues. Table S4: Transitions of amino acid residues on avian-human characteristic sites. Table S5: Transitions of amino acid residues on swine-human characteristic sites. Table S6: Top 20 sites in PB2 and their amino acid residues. (DOC) [file pone.0084638.s003.doc]

**Table S1. Catalogue of reported domains in 9 internal proteins**

| Protein | Position | Domain | References^*^ |
| --- | --- | --- | --- |
| PB2 | 196-210 | I-Aᵇ Binding Motif | (1) |
|  | 590-591 | SR Polymorphism | (2) |
|  | 449-495, 736-739 | Nuclear Localization Signal | (3) |
|  | 1-269, 580-683 | NP Binding | (4) |
|  | 1-131, 580-759 | PB1 Binding | (4) |
|  | 242-282, 538-577 | RNA cap Binding | (5) |
|  | 363-404 | RNA cap Binding | (6) |
| PB1 | 430-438, 482-490 | Epitope Region | (7) |
|  | 1-25 | PA Binding | (8) |
|  | 600-747 | PB2 Binding | (8) |
|  | 181-195, 203-252 | Nuclear Localization Signal | (9) |
|  | 1-83, 233-249, 494-757 | vRNA Binding | (10) |
|  | 1-139, 267-493 | cRNA Binding | (11) |
| PB1-F2 | 46-87 | Mitochondrial Targeting Sequence | [35] |
|  | 62-70 | H-2 D^b^ Binding Peptide | [36] |
| PA | 273-282 | B7 Binding Motif | [19] |
|  | 333-341 | K ͩ Binding Motif | [20] |
|  | 1-85, 124-139, 186-247 | Proteolysis | [21] |
|  | 124-139, 186-247 | Nuclear Localization Signal | [22] |
|  | 408-412, 668-692, 618-643, 706-716 | PB1 Binding | (8)(12) |
| NP | 91-120 | I-Aᵇ Binding Motif | (1) |
|  | 257-498 | PB2 interaction domain | (13) |
|  | 3-13, 198-216 | Nuclear Localization Signal | (14) |
| M1 | 1-164 | Membrane binding region | [24] |
|  | 165-252 | RNP Binding region | [25] |
|  | 101-105 | Nuclear Localization Signal | (15) |
|  | 89-164 | NS2 Binding | (16) |
| M2 | 1-23 | M2e Extracellular domain | [26][27] |
|  | 26-44 | Trans membrane | [26][28] |
|  | 46-62 | Amphipathic-helix | [28] |
|  | 70-73 | M1 binding | [29] |
|  | 69-84 | Epitope Region | (17) |
|  | 70-78 | A11 Binding Motif | [19] |
| NS1 | 1-73 | RNA Binding | (18)(19) |
|  | 75-79 | Linker domain | (20) |
|  | 202-230 | Disordered tail | (21) |
|  | 89-93, 164-167, 213-216 | SH2/SH3 Binding | (22)(23) |
|  | 138-147 | Nuclear export | (24) |
|  | 81-113 | eIF4GI Binding | (25)(26) |
|  | 184-186 | CPSF Binding | (26)(27) |
|  | 223-230 | PABII Binding | (26)(27) |
|  | 227-230 | PDZ ligand motif | (28) |
|  | 34-38, 216-221 | Nuclear Localization Signal | (29) |
|  | 106-115, 163-171 | B7 Binding Motif | [19] |
|  | 120-136 | Epitope Region | [34] |
| NS2 | 19-35, 85-101 | Epitope Region | [34] |
|  | 102-109 | Epitope Region | (30) |
|  | 12-21, 81-100 | Nuclear export | (31)(32) |
|  | 54-121 | M1 Binding | (17) |

^*^Citations in parentheses (e.g. (10)) are references listed below; citations in brackets (e.g. [35]) are references listed with the main text.

**References**

(1) Crowe SR, Miller SC, Brown DM, Adams PS, Dutton RW, et al. (2006) Uneven distribution of MHC class II epitopes within the influenza virus. Vaccine 24(4): 457-467

(2) Mehle A, Doudna JA (2009) Adaptive strategies of the influenza virus polymerase for replication in humans. Proc Natl Acad Sci U S A. 106(50):21312-6.

(3) Mukaigawa J, Nayak DP (1991) Two signals mediate nuclear localization of influenza virus (A/WSN/33) polymerase basic protein 2. J Virol 65(1): 245-253.

(4) Poole E, Elton D, Medcalf L, Digard P (2004) Functional domains of the influenza A virus PB2 protein: identification of NP- and PB1-binding sites. Virology 321(1): 120-133.

(5) Honda A, Mizumoto K, Ishihama A (1999) Two separate sequences of PB2 subunit constitute the RNA cap-binding site of influenza virus RNA polymerase. Genes Cells 4(8): 475-485.

(6) Fechter P, Mingay L, Sharps J, Chambers A, Fodor E, et al. (2003) Two aromatic residues in the PB2 subunit of influenza A RNA polymerase are crucial for cap binding. J Biol Chem 278: 20381-20388.

(7) Ichihashi T, Yoshida R, Sugimoto C, Takada A, Kajino F (2011) Cross-Protective Peptide Vaccine against Influenza A Viruses Developed in HLA-A*2402 Human Immunity Model. PLoS ONE 6(9): e24626.

(8) Ohtsu Y, Honda Y, Sakata Y, Kato H, Toyoda T (2002) Fine mapping of the subunit binding sites of influenza virus RNA polymerase. Microbiol Immunol 46: 167-175.

(9) Nath ST, Nayak DP (1990) Function of two discrete regions is required for nuclear localization of polymerase basic protein 1 of A/WSN/33 influenza virus (H1 N1). Mol Cell Biol. 10(8): 4139-4145.

(10) Jung TE, Brownlee GG (2006) A new promoter-binding site in the PB1 subunit of the influenza A virus polymerase. J Gen Virol 87: 679-688.

(11) Gonzalez S, Ortín J (1999) Distinct regions of influenza virus PB1 polymerase subunit recognize vRNA and cRNA templates. EMBO J 18: 3767-3775.

(12) He X, Zhou J, Bartlam M, Zhang R, Ma J, et al. (2008) Crystal structure of the polymerase PA(C)-PB1(N) complex from an avian influenza H5N1 virus. Nature 454(7208): 1123-1126.

(13) Biswas SK, Boutz PL, Nayak DP (1998) Influenza Virus Nucleoprotein Interacts with Influenza Virus Polymerase. ProteinsJ Virol.72(7): 5493-5501.

(14) Ozawa M, Fujii K, Muramoto Y, Yamada S, Yamayoshi S, et al. (2007) Contributions of two nuclear localization signals of influenza A virus nucleoprotein to viral replication. J Virol 81(1): 30-41.

(15) Z Ye, Robinson D, Wagner RR (1995) Nucleus-Targeting Domain of the Matrix Protein (M1) of Influenza Virus. J Virol 69(3): 1964-1970.

(16) Akarsu H, Burmeister WP, Petosa C, Petit I, Mu¨ller CW, et al. (2003) Crystal structure of the M1 protein-binding domain of the influenza A virus nuclear export protein (NEP/NS2). EMBO J 22(18): 4646-4655.

(17) Lee LY, Ha do LA, Simmons C, de Jong MD, Chau NV, et al. (2008) Memory T cells established by seasonal human influenza A infection cross-react with avian influenza A (H5N1) in healthy individuals. J Clin Invest, 118(10): 3478-3490.

(18) Hale BG, Randall RE, Ortín J, Jackson D (2008) The multifunctional NS1 protein of influenza A viruses. J Gen Virol 89(10): 2359-76.

(19) Qian XY, Chien CY, Lu Y, Montelione GT, Krug RM (1995) An aminoterminal polypeptide fragment of the influenza virus NS1 protein possesses specific RNA-binding activity and largely helical backbone structure. RNA 1(9): 948-956.

(20) Bornholdt ZA, Prasad BV (2008) X-ray structure of NS1 from a highly pathogenic H5N1 influenza virus. Nature 456(7224): 985-8.

(21) Hale BG, Barclay WS, Randall RE, Russell RJ (2008) Structure of an avian influenza A virus NS1 protein effector domain. Virology. 378(1): 1-5.

(22) Heikkinen LS, Kazlauskas A, Melén K, Wagner R, Ziegler T, et al. (2008) Avian and 1918 Spanish influenza a virus NS1 proteins bind to Crk/CrkL Src homology 3 domains to activate host cell signaling. J Biol Chem. 283(9): 5719-27.

(23) Shin YK, Liu Q, Tikoo SK, Babiuk LA, Zhou Y (2007) Influenza A virus NS1 protein activates the phosphatidylinositol 3-kinase (PI3K)/Akt pathway by direct interaction with the p85 subunit of PI3K. J Gen Virol 88: 13-18.

(24) Li Y, Yamakita Y, Krug RM (1998) Regulation of a nuclear export signal by an adjacent inhibitory sequence: the effector domain of the influenza virus NS1 protein. Proc Natl Acad Sci USA 95(9): 4864-4869.

(25) Aragon T, de la Luna S, Novoa I, Carrasco L, Ortín J, et al. (2000) Eukaryotic translation initiation factor 4GI is a cellular target for NS1 protein, a translational activator of influenza virus. Mol Cell Biol 20(17): 6259-6268.

(26) Kochs G, Garcia-Sastre A, Martinez-Sobrido L (2007) Multiple anti-interferon actions of the influenza A virus NS1 protein. J Virol 81(13): 7011-7021.

(27) Chen Z, Li Y, Krug RM (1999) Influenza A virus NS1 protein targets poly(A)- binding protein II of the cellular 3’-end processing machinery. EMBO J 18(8): 2273-2283.

(28) Obenauer JC, Denson J, Mehta PK, Su X, Mukatira S, et al. (2006) Large-scale sequence analysis of avian influenza isolates. Science 311(5767): 1576-1580.

(29) Greenspan D, Palese P, Krystal M (1988) Two nuclear location signals in the influenza virus NS1 nonstructural protein. J Virol. 62(8): 3020-3026.

(30) Assarsson E, Bui HH, Sidney J, Zhang Q, Glenn J, et al. (2008) Immunomic analysis of the repertoire of T-cell specificities for influenza A virus in humans. J Virol. 82(24): 12241-12251.

(31) Iwatsuki-Horimoto K, Horimoto T, Fujii Y, Kawaoka Y (2004) Generation of influenza A virus NS2 (NEP) mutants with an altered nuclear export signal sequence. J Virol 78(18): 10149-10155.

(32) Shimizu T, Takizawa N, Watanabe K, Nagata K, Kobayashi N (2010) Crucial role of the influenza virus NS2 (NEP) C-terminal domain in M1 binding and nuclear export of vRNP. FEBS Lett. 585(1): 41-46.

Table S2. NS1’s chronological genomic signatures identified in 6 periods and their amino acid residues

|  |  | **Avian** | **Human** |  |  | **Avian** | **Human** |
| --- | --- | --- | --- | --- | --- | --- | --- |
| **Period** | **Position** | **AA (percent)** | **AA (percent)** | **Period** | **Position** | **AA (percent)** | **AA (percent)** |
| **1902**–**1908** | 27 | M (100%) | L (100%) | **1969**–**1977(cont.)** | 84 | VS (65% 33%) | TA (60% 32%) |
|  | 44 | G (100%) | R (100%) |  | 226 | IV (60% 35%) | A (100%) |
|  | 70 | E (100%) | K (100%) |  | 53 | D (91%) | ND (73% 23%) |
|  | 84 | A (100%) | V (100%) |  | 70 | EK (57% 37%) | K (100%) |
|  | 129 | T (100%) | I (100%) | **1978**–**2009** | 60 | AE (54% 42%) | V (96%) |
|  | 136 | I (100%) | V (100%) |  | 114 | SG (66% 24%) | P (98%) |
|  | 178 | I (100%) | V (100%) |  | 125 | D (95%) | ED (77% 23%) |
|  | 209 | D (100%) | N (100%) |  | 48 | S (94%) | NS (76% 23%) |
|  | 210 | R (100%) | G (100%) |  | 227 | E (94%) | R (99%) |
|  | 227 | E (100%) | K (100%) |  | 70 | EK (66% 30%) | K (98%) |
|  | 216 | TP (67% 33%) | P (100%) |  | 81 | I (95%) | MI (65% 34%) |
| **1919**–**1957** | 70 | E (100%) | K (97%) |  | 171 | DT (62% 23%) | IY (43% 33%) |
|  | 22 | F (91%) | V (100%) |  | 59 | RM (60% 24%) | HL (43% 33%) |
|  | 114 | S (91%) | P (100%) |  | 209 | DN (65% 32%) | N (79%) |
|  | 227 | E (100%) | R (100%) |  | 21 | RL (74% 26%) | QR (66% 34%) |
|  | 3 | S (100%) | P (97%) |  | 215 | P (78%) | TP (65% 34%) |
|  | 81 | IT (73% 27%) | M (100%) |  | 22 | FL (72% 26%) | VF (64% 34%) |
|  | 215 | P (90%) | T (100%) |  | 112 | AT (56% 42%) | EI (43% 32%) |
|  | 209 | D (70%) | N (100%) |  | 67 | RD (70% 23%) | KW (43% 33%) |
|  | 224 | R (100%) | GR (67% 33%) |  | 18 | VI (73% 27%) | IV (76% 23%) |
|  | 27 | ML (55% 45%) | L (100%) |  | 229 | E (97%) | KE (66% 33%) |
| **1958**–**1968** | 215 | P (100%) | T (100%) |  | 129 | IT (65% 31%) | MV (39% 32%) |
|  | 227 | E (100%) | R (100%) |  | 166 | LM(69% 25%) | FL (60% 40%) |
|  | 229 | E (100%) | K (100%) | **2010**–**2013** | 125 | D (100%) | E (99%) |
|  | 53 | D (100%) | N (100%) |  | 48 | S (97%) | N (100%) |
|  | 81 | I (100%) | M (100%) |  | 60 | AE (59% 33%) | V (98%) |
|  | 196 | E (100%) | K (100%) |  | 114 | SG (73% 21%) | P (94%) |
| **1969**–**1977** | 81 | I (100%) | M (92%) |  | 67 | R (78%) | WK (75% 22%) |
|  | 119 | M (100%) | I (92%) |  | 18 | VI (79% 21%) | I (98%) |
|  | 196 | E (100%) | K (92%) |  | 74 | D (98%) | SD (74% 24%) |
|  | 215 | P (96%) | T (92%) |  | 59 | R (68%) | LH (75% 22%) |
|  | 227 | E (100%) | R (100%) |  | 112 | AT (62% 35%) | IE (75% 23%) |
|  | 229 | E (100%) | K (100%) |  | 189 | D (98%) | GD (74% 24%) |
|  | 21 | RL (67% 33%) | Q (92%) |  | 78 | K (100%) | RK (71% 29%) |
|  | 129 | I (78%) | M (92%) |  | 70 | EK (74% 23%) | K (95%) |
|  | 98 | MI (65% 35%) | L (92%) |  | 197 | TN (72% 26%) | N (99%) |
|  | 112 | AT (65% 35%) | E (92%) |  | 178 | I (99%) | VI (73% 27%) |
|  | 60 | AE (63% 31%) | V (92%) |  | 26 | ED (79% 20%) | G (77%) |
|  | 23 | AS (67% 31%) | V (88%) |  | 119 | M (99%) | LM (73% 27%) |
|  | 171 | DT (61% 33%) | I (92%) |  | 171 | DT (67% 21%) | YI (71% 23%) |
|  | 56 | TV (63% 31%) | A (92%) |  | 217 | K (92%) | EK (75% 24%) |
|  | 22 | FL (63% 33%) | V (85%) |  | 209 | DN (76% 24%) | N (94%) |
|  | 114 | SG (62% 35%) | P (92%) |  | 25 | Q (80%) | NQ (75% 23%) |

**Table S3. M1’s chronological genomic signatures identified in 6 periods and their amino acid residues**

|  |  | **Avian** | **Human** |
| --- | --- | --- | --- |
| **Period** | **Position** | **AA (percent)** | **AA (percent)** |
| **1902**–**1918** | 101 | K (100%) | R (100%) |
|  | 121 | T (100%) | A (100%) |
|  | 144 | L (100%) | F (100%) |
|  | 234 | L (100%) | I (100%) |
| **1919**–**1957** | 121 | T (100%) | A (100%) |
|  | 137 | T (100%) | A (100%) |
|  | 115 | V (100%) | I (100%) |
|  | 218 | T (100%) | AT (57% 43%) |
|  | 219 | VI (54% 46%) | I (87%) |
|  | 15 | IV (64% 36%) | V (83%) |
| **1958**–**1968** | 218 | T (100%) | A (86%) |
|  | 137 | T (100%) | A (93%) |
|  | 115 | V (94%) | I (93%) |
|  | 121 | TA (63% 38%) | A (100%) |
| **1969**–**1977** | 121 | T (95%) | A (100%) |
|  | 218 | T (100%) | A (94%) |
|  | 115 | V (100%) | I (94%) |
|  | 137 | T (100%) | A (83%) |
|  | 167 | T (100%) | AT (61% 39%) |
| **1978**–**2009** | 115 | V (98%) | IV (69% 30%) |
|  | 137 | T (96%) | AT (69% 31%) |
|  | 121 | T (83%) | AT (70% 30%) |
|  | 218 | T (95%) | AT (59% 39%) |
|  | 227 | A (91%) | TA (62% 37%) |
| **2010**–**2013** | 214 | Q (100%) | H (86%) |
|  | 209 | A (99%) | T (86%) |
|  | 116 | A (100%) | S (85%) |
|  | 30 | D (99%) | S (76%) |
|  | 207 | S (84%) | N (85%) |
|  | 142 | VG (64% 33%) | A (84%) |

**Table S4. Transitions of amino acid residues on avian-human characteristic sites**

| **Protein** | **Position^a^** | **Avian** | **Human** | **Protein** | **Position^a^** | **Avian** | **Human** |
| --- | --- | --- | --- | --- | --- | --- | --- |
|  |  | AA Transition^b^ | AA Transition^b^ |  |  | AA Transition^b^ | AA Transition^b^ |
| **PB2** | 9 | D=D=D=D=D=D | D→N=N=N→ND→DN | **PA(cont.)** | 404 | A=A=A=A=A=A | A→SA→S=S→SA→AS |
|  | 44 | A=A=A=A=A=A | A→SA→S=S→SA→AS |  | 421 | S→SG→S=S=S=S | S→IS→IT→I→ISV→SV |
|  | 64 | M=M=M=M=M=M | M→T=T=T→TM→MT |  | 552 | T=T=T=T=T=T | S=S=S=S→ST→TS |
|  | 81 | T=T=T=T=T=T | T→M=M=M→MT→T |  | 668 | I=I=I=I=I=I | I→IV→V=V→IV=IV |
|  | 82 | N=N=N=N=N=N | N→NS→S=S→NS=NS |  | 682 | N→D=D=D=D=D | D=D=D=D=D=D |
|  | 105 | T=T=T=T=T=T | T→M=M→VM→VT→TV |  | 716 | K=K→KR→K=K=K | R=R=R=R→K=K |
|  | 108 | T=T=T=T=T=T | A→T=T=T=T=T |  | **85** | T=T=T=T=T=T | T=T=T=T→TI→IT |
|  | 114 | V=V=V=V=V=V | I=I→VI→V=V=V |  | **186** | G=G=G=G=G=G | G=G=G=G→GS→SG |
|  | 122 | I→V=V=V=V=V | V=V=V=V=V=V |  | **204** | R=R=R=R=R=R | R=R=R=R→K=K |
|  | 134 | R→H=H=H=H=H | H=H=H=H=H=H |  | **213** | R=R=R=R=R=R | R→RK→R=R→RK→KR |
|  | 156 | S→A=A=A=A=A | A=A=A=A=A=A |  | **256** | R=R=R=R=R=R | R→RK→K→RK→K→KQ |
|  | 199 | A=A=A=A=A=A | S=S=S=S→SA→AS |  | **262** | K=K=K=K=K=K | K=K=K=K→KR→RK |
|  | 368 | R=R=R=R=R=R | R→KR→K=K→KR→RK |  | **275** | P=P=P=P=P=P | P=P=P=P→PL→LP |
|  | 382 | I=I=I=I=I=I | I=I→VI→V→IV=IV |  | **277** | S=S=S=S=S=S | S=S→FS→F→YH→HY |
|  | 461 | V→I=I=I=I=I | I=I=I→VI→IV→I |  | **323** | V=V=V→VI=VI→IV | V=V=V=V=V=V |
|  | 463 | I=I=I=I=I=I | I→IV→V=V→IV=IV |  | **336** | L=L=L=L=L=L | L=L=L=L→LM→ML |
|  | 470 | N→S=S=S=S=S | S=S=S=S=S=S |  | **356** | K=K=K=K=K=K | K→RK→R=R=R=R |
|  | 475 | L=L=L=L=L=L | M=M=M=M→ML→LM |  | **362** | K=K=K=K=K=K | K=K=K=K→KR→RK |
|  | 491 | T=T=T=T=T=T | T→A→T=T→TA→T |  | **407** | I=I=I=I=I=I | I=I=I=I→IV→VI |
|  | 539 | I=I=I=I=I=I | V→I=I=I=I=I |  | **409** | S=S=S=S=S=S | S→N=N=N=N=N |
|  | 567 | D→DV→D=D=D=D | N=N=N=N→ND→DN |  | *382* | E=E=E=E=E=E | D=D=D=D=D→DE |
|  | 613 | V=V=V=V=V=V | V→TA→T=T→TV→V | **NP** | 16 | G=G=G=G=G=G | D=D=D=D→DG→GD |
|  | 627 | E=E=E=E=E=E | K=K=K=K→KE→EK |  | 31 | R=R=R=R=R=R | R→K=K=K→KR→RK |
|  | 661 | A=A=A=A=A=A | A→T=T=T→TA→AT |  | 34 | GS=GS→G=G=G=G | G→DG→D=D→DG→GD |
|  | 667 | V=V=V=V=V=V | V→VI→I=I→VI=VI |  | 61 | I=I=I=I=I=I | I→L=L=L→LI→IL |
|  | 674 | A=A=A=A=A=A | A→T=T=T→TA→AT |  | 98 | RK→R=R=R=R=R | R→KR→K=K→RK→R |
|  | 680 | N→DG→D=D=D=D | D=D=D=D=D=D |  | 105 | VM=VM→M→MV=MV=MV | M=M=M=M=M=M |
|  | 702 | K=K=K=K=K=K | R=R=R=R→RK→KR |  | 109 | IT→I=I=I=I=I | I→VI→V=V→VI→IV |
|  | **54** | K=K=K=K=K=K | K=K=K=K→KR→RK |  | 127 | E=E=E=E=E=E | E→D=D=D→ED→E |
|  | **65** | E=E=E=E=E=E | E=E=E=E→ED→DE |  | 146 | A=A=A=A=A=A | A→TA→T=T→AT→A |
|  | **147** | I=I=I=I=I=I | I=I=I=I→IT→TI |  | 214 | RK→R=R=R=R=R | R→K=K=K→KR→RK |
|  | **184** | T=T=T=T=T=T | T=T=T=T→TA→AT |  | 283 | LI→L=L=L=L=L | P=P=P=P→PL→LP |
|  | **225** | S=S=S=S=S=S | S=S=S=S→SG→GS |  | 293 | RK→R=R=R=R=R | R→KR→K=K→KR→RK |
|  | **292** | I=I=I=I=I=I | I→TI→T=T→TV→VT |  | 334 | H=H=H=H=H=H | H→NH→N→NH→H=H |
|  | **315** | M=M=M=M=M=M | M=M=M=M→MI→IM |  | 372 | E=E=E=E=E=E | E→DE→D=D→DE→ED |
|  | **340** | R=R=R=R→RK=RK | R=R=R=R→KR→K |  | 375 | D=D=D=D=D=D | D→E=E=E→GD→DG |
|  | **453** | P=P=P=P=P=P | P→HP→H=H→HS→SH |  | 421 | E=E=E=E=E=E | E→D=D=D→E=E |
|  | **559** | T=T=T=T=T=T | T=T=T=T→IAT→IA |  | 422 | R=R=R=R=R=R | R→KR→K=K→KR→RK |
|  | **588** | A=A=A=A=A=A | A→I=I=I→IT→TI |  | 423 | A=A=A=A=A=A | A→TP→P→SP→SA→AS |
|  | **590** | G=G=G=G=G=G | G=G=G=G→SG→S |  | 442 | T=T=T=T=T=T | T→AT→A=A→AT→TA |
|  | **591** | Q=Q=Q=Q=Q=Q | Q=Q=Q=Q→QR→RQ |  | 455 | D=D=D=D=D=D | D→ED→E=E→ED→DE |
|  | **645** | M=M=M=M=M=M | M=M=M=M→ML→LM |  | 473 | N→NS→N=N=N=N | S→NS→N=N=N=N |
|  | **684** | A=A=A=A=A=A | A→AS→S=S→SA→S |  | **189** | M=M=M=M=M=M | M=M=M=M→MI→IM |
|  | *271* | A→T=T=T=T=T | T→A=A=A=A=A |  | **190** | V=V=V=V=V=V | V=V=V=V→VA→AV |
| **PB1** | 54 | K=K=K=K=K=K | R→RK→K=K→KR→K |  | **217** | VI→I=I=I=I=I | I=I→S→GS→SV→VS |
|  | 108 | L=L=L=L=L=L | L→IL→L=L→LI→L |  | **289** | Y=Y=Y=Y=Y=Y | Y=Y=Y=Y→YH→HY |
|  | 114 | V=V=V=V=V=V | V→VI→IV→V=V=V |  | **305** | RK→R=R=R=R=R | R→KR→K=K=K=K |
|  | 121 | K=K=K=K=K=K | K=K→KR→RK→K=K |  | **351** | R=R=R=R=R=R | R→K=K=K=K=K |
|  | 171 | M=M=M=M=M=M | M→MI→IM→M=M=M |  | **350** | T=T=T=T=T=T | T=T=T=T→TK→KT |
|  | 212 | L=L=L=L=L=L | L=L→LV→V→LV=LV |  | **353** | V=V=V=V=V=V | V→IV→S=S→SI→IS |
|  | 261 | S=S=S=S=S=S | S=S→NS→S=S=S |  | **400** | KR→R=R=R=R=R | R=R=R=R→RK→KR |
|  | 327 | R=R=R=R=R=R | R=R→RK→K→KR→RK |  | **425** | I=I=I=I=I=I | I=I=I=I→VI=VI |
| **PB1(cont.)** | 349 | G→A=A=A=A=A | A=A=A=A=A=A | **NP(cont.)** | **430** | T=T=T=T=T=T | T=T=T=T→TS→ST |
|  | 375 | S=S→SN→NS=NS=NS | S→SN→S=S=S=S |  | **433** | T=T=T=T=T=T | T→AT→T=T→TN→NT |
|  | 383 | E=E→ED→E=E=E | D→DE→E=E=E=E |  | **444** | I=I=I=I=I=I | I=I=I=I→IV→VI |
|  | 384 | P→S=S=S=S=S | S=S=S=S=S=S |  | **452** | R=R=R=R=R=R | R→RK→K=K=K=K |
|  | 396 | I→L=L=L=L=L | L=L=L=L=L=L |  | **456** | V=V=V=V=V=V | V=V→M→V→VL→LV |
|  | 401 | A=A=A=A=A=A | A→AV→VA→A=A=A |  | *100* | R=R=R=R=R=R | I→V=V=V→VI→IV |
|  | 464 | D=D=D=D=D=D | D=D→ND→D=D=D |  | *136* | L=L=L=L=L=L | M=M=M=M→IM→I |
|  | 473 | V=V=V=V=V=V | L→LV→V=V→VL→V |  | *313* | F=F=F=F=F=F | Y=Y=Y=Y→YV→VY |
|  | 576 | L=L=L=L=L=L | I→IL→L=L→LI=LI |  | 33 | V=V=V=V=V=V | I=I=I=I=I=I |
|  | 596 | P=P=P=P=P=P | P=P→SP→P=P=P |  | 357 | Q=Q=Q=Q=Q=Q | K=K=K=K=K=K |
|  | 628 | M→L=L=L=L=L | L=L=L=L=L=L | **M1** | 15 | ILV→IV→V=V→VI→IV | V=V=V=V→VI→I |
|  | 645 | V=V=V=V=V=V | M→MV→V=V=V=V |  | 101 | K→RK→R=R→RK→KR | R=R=R=R→RK→K |
|  | 654 | S=S=S=S=S=S | N→NS→S=S→SN→S |  | 115 | V=V=V=V=V=V | V→I=I=I→IV→V |
|  | 667 | I=I=I=I=I=I | I→IV→TI→I→IV→I |  | 121 | T=T→TA→T=T=T | A=A=A=A→AT→T |
|  | 691 | K=K=K=K=K=K | K→RK→K=K→KR→K |  | 137 | T=T=T=T=T=T | T→A=A=A→AT→T |
|  | **12** | V=V=V=V=V=V | V=V=V=V→VI→IV |  | 144 | L→FL→F=F→FL=FL | F=F=F=F=F=F |
|  | **175** | D=D=D=D=D=D | D→DN→D=D→DN→ND |  | 167 | T=T=T=T=T→TA | T=T=T→AT→TA→T |
|  | **179** | M=M=M=M=M=M | M=M=M=M→IM=IM |  | 218 | T=T=T=T=T=T | T→AT→A=A→AT→T |
|  | **216** | S=S=S=S=S=S | S=S=S=S→GS→G |  | 219 | IV→VI→I=I=I→IV | I=I=I=I=I=I |
|  | **298** | L=L=L=L=L=L | L→LI→L=L→IL=IL |  | 227 | A=A=A=A=A→AT | A=A=A=A→TA→A |
|  | **336** | V=V=V=V=V=V | V=V=V→IV→I=I |  | 234 | L=L=L=L→LI=LI | I→L=L=L=L=L |
|  | **339** | I=I=I=I=I=I | I=I=I=I→IM→MI |  | **30** | D=D=D=D=D=D | D=D=D=D→DS→S |
|  | **361** | S=S=S=S=S=S | S=S=S→RS=RS→R |  | **116** | A=A=A=A=A=A | A=A=A=A→AS→S |
|  | **364** | L=L=L=L=L=L | L=L=L=L→LI→IL |  | **142** | V=V=V=V=V→VG | V=V=V=V→VAS→A |
|  | **430** | R=R=R=R=R→RK | R→RK→KR=KR=KR→K |  | **207** | S=S=S=S=S=S | S=S=S=S→SN→N |
|  | **486** | R=R=R=R=R=R | R=R=R=R→KR→K |  | **209** | A=A=A=A=A=A | A=A=A=A→AT→T |
|  | **517** | I=I=I=I=I=I | I=I=I=I→VI=VI |  | **214** | Q=Q=Q=Q=Q=Q | Q=Q=Q=Q→QH→H |
|  | **581** | E=E=E=E=E=E | E=E=E=E→D=D | **M2** | 11 | T=T=T=T=T=T | T→I=I=I→IT→TI |
|  | **584** | R=R=R=R=R=R | R=R=R=R→Q=Q |  | 16 | E=E=E=E=E=E | G=G=G=G→GE→EG |
|  | **587** | A=A=A=A=A=A | A=A=A=A→AV→V |  | 20 | S=S=S=S=S=S | N=N=N=N→NS→SN |
|  | **618** | E=E=E=E=E=E | E=E=E=E→ED→DE |  | 28 | I=I=I=I→IV=IV | I→VI→V=V→VI→IV |
|  | **621** | Q=Q=Q=Q=Q=Q | Q=Q=Q=Q→RQ→R |  | 54 | R→RC→R=R=R=R | R→LRF→F=F→LIR→RL |
|  | **638** | E=E=E=E=E=E | E=E=E=E→ED→DE |  | 55 | L=L=L=L→LF→FL | L→F→FL→F=F=F |
|  | **728** | I=I=I=I=I=I | I=I=I=I→IV→VI |  | 56 | K=K=K=K=K=K | K=K→EK→E→K=K |
|  | **741** | A=A=A=A=A=A | A→TA→A→AS→S=S |  | 57 | Y=Y=Y=Y=Y=Y | Y→HY→H=H→HY→YH |
| **PB1-F2** | 2 | →E=E=E=E=E | →GE=GE→E→EG→E |  | 78 | Q=Q=Q=Q=Q=Q | K=K=K=K→KQE→QK |
|  | 10 | →T→TI→T=T=T | →IT→T=T→TI→T |  | 82 | N→NS→S=S=S→SN | S=S=S=S→NS→SN |
|  | 11 | →Q=Q=Q=Q→QR | →LQ→Q=Q=Q=Q |  | 86 | V=V=V=V=V=V | V→A=A=A→AV→VA |
|  | 16 | →IT→I=I→IT=IT | →I→IT→I→TI→T |  | 89 | G=G=G=G=G=G | G→SG→S=S→SG→GS |
|  | 17 | →N=N=N=N=N | →SN→N=N→NS→N |  | **18** | R→RS→KR=KR=KR→KRN | R=R=R=R=R=R |
|  | 22 | →G→EG→GE=GE→G | →EG→G=G→GE→G |  | **31** | NS→S=S=S=S→SN | S→SN→S=S→SN→N |
|  | 23 | →NS→N→NS=NS=NS | →DS→S=S→SD→SN |  | **43** | L=L=L=L=L=L | L=L=L=L→LIT→TL |
|  | 28 | →R→LRQ→QR→Q=Q | →PR→R→QR→QP→Q |  | **77** | R=R=R=R=R=R | R=R=R=R→RQ→QR |
|  | 29 | →KR→K→KR→RK=RK | →K=K→RK→K→KR |  | **93** | N=N=N=N=N=N | N→SN→S=S→SN=SN |
|  | 32 | →RH→HR=HR→H=H | →HR→R=R→H→HR |  | 14 | G=G=G=G=G=G | E=E=E=E=E=E |
|  | 33 | →P→PH→P=P=P | →HP→P=P→PR→P | **NS1** | 3 | S=S=S=S=S=S | S→P→S=S=S=S |
|  | 35 | →S→SL→LS→SL=SL | →SL→L=L→S=S |  | 21 | R=R→RL=RL=RL=RL | R=R→Q=Q→QR→RQ |
|  | 43 | →L→LP=LP→L=L | →QL→L=L=L=L |  | 22 | F=F→FL=FL=FL=FL | F→V=V=V→VF→FV |
|  | 44 | →R→RK→R=R=R | →KR→R=R→RK→R |  | 23 | A=A→AS=AS=AS→A | A=A→VA→V→AV=AV |
|  | 50 | →D→DVG→D=D→DG | →VD→D=D→DA→D |  | 27 | M→ML→LM=LM=LM=LM | L=L=L=L=L=L |
|  | 52 | →H→HL→H=H=H | →PH→H=H→HP→H |  | 44 | G→RG→RK=RK=RK=RK | R=R=R=R→KR=KR |
|  | 55 | →T=T→TI=TI=TI | →IT→T=T→TI→T |  | 53 | DH→D=D=D=D=D | D→DN→N→ND→DN→D |
|  | 56 | →A→VA=VA→V=V | →VA→A→V=V=V |  | 56 | T=T→TV=TV=TV→T | T=T→AT→A→TA=TA |
|  | 57 | →S→SC→S=S→SY | →YS→S=S→SY→S |  | 81 | I→IT→I=I=I=I | I→M=M=M→MI→IM |
|  | 65 | →K→KR→K=K=K | →RK→K=K=K=K |  | 84 | A→VA→VS=VS=VS→V | V→VA→A→TA→TVA→VT |
|  | 66 | →N→NS=NS→SN=SN | →N=N=N=N=N |  | 98 | M=M→MI=MI=MI=MI | M=M→LM→L→ML=ML |
| **PB1-F2(cont.)** | 67 | →P=P=P=P=P | →P→HP→P=P=P | **NS1(cont.)** | 129 | T→IT→I=I→IT=IT | I=I→MI→M→MVI→V |
|  | 69 | →QP=QP→Q=Q=Q | →L→Q=Q=Q=Q |  | 136 | I→VI→V=V=V=V | V=V=V=V=V=V |
|  | 74 | →TI→T=T=T=T | →T→IT→T=T=T |  | 166 | L=L→LM=LM=LM=LM | L=L=L→FL=FL→LF |
|  | 81 | →K=K=K=K=K | →RK→K=K=K=K |  | 196 | E=E=E=E=E=E | E→EK→K=K→EK=EK |
|  | 84 | →N→SN→NS=NS=NS | →SN→N=N=N=N |  | 210 | R→GR→G=G=G=G | G=G=G=G=G=G |
|  | 86 | →Q→QH→Q=Q=Q | →HQ→Q=Q=Q=Q |  | 215 | P=P=P=P=P=P | P→T=T=T→TP→PT |
|  | 90 | →NS→N→NS=NS→N | →NS→N→D→N=N |  | 216 | TP→P=P=P=P=P | P=P=P=P=P=P |
|  | **6** | →D=D=D=D=D | →DG→D=D→GD=GD |  | 224 | R=R=R=R=R=R | R→GR→R=R→RG→R |
|  | **21** | →R=R=R=R→RK | →GR→RG→K→R→G |  | 226 | I=I=I→IV=IV→I | I=I=I→A=A=A |
|  | **25** | →Q=Q=Q=Q=Q | →Q=Q=Q→RQ→R |  | 227 | E=E=E=E=E=E | K→R=R=R=R=R |
|  | **27** | →T=T=T=T=T | →T=T=T→IT→I |  | 229 | E=E=E=E=E=E | E=E→K=K→KE=KE |
|  | **31** | →E→EG→E=E=E | →E=E→G→GE→G |  | **18** | V=V→VI=VI=VI=VI | V=V=V=V→IV→I |
|  | **34** | →N=N=N=N=N | →N=N=N=N→S |  | **25** | Q=Q→QR=QR=QR→Q | Q=Q=Q=Q→QN→NQ |
|  | **42** | →YC=YC→Y→CY=CY | →YC→Y=Y=Y=Y |  | **26** | E=E→ED=ED=ED=ED | E=E=E=E→EG→G |
|  | **45** | →T→TI→IT→TI→T | →T=T→I→IT→I |  | **48** | S=S=S=S=S=S | S=S=S=S→NS→N |
|  | **47** | →NS=NS→SN→S=S | →N=N→S→SN→N |  | **59** | R=R→RM=RM=RM→R | R=R=R→HR→HLC→LH |
|  | **59** | →K→KR=KR→K=K | →K=K=K→R=R |  | **60** | A=A→AE=AE=AE=AE | A→AV→V=V=V=V |
|  | **60** | →Q=Q=Q=Q=Q | →Q=Q=Q→LP→L |  | **67** | RP→R→RD=RD=RD→R | R=R=R→KR→KWR→WK |
|  | **62** | →L=L=L=L=L | →L=L=L→PL→P |  | **70** | E=E→EK=EK=EK=EK | K=K=K=K=K=K |
|  | **70** | →E→EV→GE→EG=EG | →VE→E→G→GV→V |  | **74** | D=D=D=D=D=D | D=D=D=D→DS→SD |
|  | **73** | →K=K=K=K=K | →K=K=K→R=R |  | **78** | K=K=K=K=K=K | K=K=K=K→KR→RK |
|  | **75** | →RH→R=R=R=R | →R=R=R→H=H |  | **112** | A=A→AT=AT=AT=AT | A=A→EA→E→EIA→IE |
|  | **76** | →V=V=V=V=V | →V=V→AV→A=A |  | **114** | S=S→SG=SG=SG=SG | S→P=P=P=P=P |
|  | **79** | →R=R=R=R=R | →RQ→R=R→Q=Q |  | **119** | M=M=M=M=M=M | M=M→IM→I→ML→LM |
|  | **82** | →L→SL→L=L=L | →L=L→LS→SP→P |  | **125** | D=D=D=D=D=D | D=D=D=D→ED→E |
|  | **85** | →K=K=K=K=K | →K=K=K→KR→R |  | **171** | D=D→DT=DT=DT=DT | D→NDA→IN→I→IYN→YI |
|  | **87** | →E→G→EG→E=E | →E=E→G=G=G |  | **178** | I=I=I=I=I=I | V→IV→I=I→IV→VI |
| **PA** | 28 | P=P=P=P=P=P | L=L=L=L→LP→PL |  | **189** | D=D=D=D=D=D | D→DN→D=D→DG→GD |
|  | 30 | L→I=I=I=I=I | I=I=I=I=I=I |  | **197** | T=T→TN=TN=TN=TN | T=T=T→TN→NT→N |
|  | 55 | D=D=D=D=D=D | N=N=N=N→ND→DN |  | **209** | D=D=D→DN=DN=DN | N=N=N=N=N=N |
|  | 57 | R=R=R=R=R=R | R→Q=Q=Q→QR→RQ |  | **217** | K=K=K=K=K=K | K=K=K=K→KET→EK |
|  | 65 | S→SP→S=S=S=S | S→L=L=L→LS→SL | **NS2** | 3 | S=S=S=S=S=S | S→P→SP→S=S=S |
|  | 66 | S→G=G=G=G=G | G→DG→D=D→DG→GD |  | 14 | M=M→MQ=MQ=MQ→M | M=M→LM→L→ML=ML |
|  | 100 | V=V=V=V=V=V | A=A=A=A→AV→VA |  | 52 | I→M=M=M=M=M | M=M=M=M=M=M |
|  | 120 | V→I=I=I=I=I | I=I=I=I=I=I |  | 60 | S→SN=SN=SN=SN=SN | N=N=N=N→NSH→SN |
|  | 142 | K=K=K=K=K=K | K=K→NK→N→KN=KN |  | 86 | R=R→RI=RI→R=R | R→KR→RK→R→RK→R |
|  | 159 | E→A=A=A=A=A | A=A=A=A=A=A |  | 107 | L=L=L=L=L=L | L→FL→F=F→FL→LF |
|  | 176 | Y→F=F=F=F=F | F=F=F=F=F=F |  | **6** | V=V→VI=VI=VI→V | V=V=V=V→VM→MV |
|  | 184 | S=S=S=S=S=S | S=S→N=N→SN=SN |  | **32** | I=I=I=I=I=I | I=I=I=I→IV→VI |
|  | 225 | S=S=S=S=S=S | S→CS→C=C→CS→SC |  | **34** | Q=Q=Q=Q=Q=Q | Q=Q=Q=Q→QR→RQ |
|  | 241 | C=C=C=C=C=C | Y=Y=Y→C=C=C |  | **40** | L=L→LI=LI=LI=LI | L=L=L=L→IL→I |
|  | 268 | L=L=L=L=L=L | L→IL→I=I→IL→LI |  | **48** | A=A=A→AS=AS→A | A=A=A=A→AT→TA |
|  | 312 | K=K=K=K=K=K | R=R=R=R→KR→K |  | **57** | S=S=S=S=S=S | S=S=S→LS→YLS→YL |
|  | 321 | N=N=N=N=N=N | N→YN→Y=Y→YN→NKY |  | **63** | G=G→GA=GA=GA→G | G=G=G=G→GE→EG |
|  | 322 | I=I=I=I=I=I | V=V→IV→I=I=I |  | **70** | S=S=S=S=S=S | S→G=G=G=G=G |
|  | 337 | A=A=A=A=A=A | A→S=S=S→SA→AS |  | **83** | V=V→VC=VC=VC→V | V=V=V=V→VM→MV |
|  | 385 | K=K=K=K=K=K | K→KR→R=R→KR=KR |  | **89** | I=I→IK→KIV→IK→I | I=I→TI→T→TA→AT |
|  | 400 | R→PQ=PQ=PQ→PS=PS | L=L=L=L→LP→PL |  | **115** | T=T=T=T=T=T | T=T=T=T→TA→AT |
|  | 403 | P→L=L=L=L=L | L=L=L=L=L=L |  |  |  |  |

^a^The position number in regular face indicates this site was a signature within specific periods before 2010, but turned to a non-signature during 2010~2013. The number in boldface indicates this site was a nonsignature within specific periods before 2010, but became a signature during 2010~2013. The position number in italic indicates this site was a chronological signature throughout all periods, but their dominant AA varied; the underlined number indicates this site was not only a chronological signature across all periods, but their dominant AA also remained constant.

^b^Transition of dominant amino acid residues on characteristic sites. Distinct dominant AA between different hosts are underlined. The symbol “→” indicates a transition of dominant amino acids between two periods, e.g. VM→VT or DE→D; the symbol “=” indicates there was no change in dominant amino acids, e.g. T=T or A=A. Because of the lack of PB1-F2 data during 1902~1918, we did not show the PB1-F2 chronological signatures during 1902~1918, as denoted by .

**Table S5. Transitions of amino acid residues on swine-human characteristic sites**

| **Protein** | **Position^a^** | **Swine** | **Human** | **Protein** | **Position^a^** | **Swine** | **Human** |
| --- | --- | --- | --- | --- | --- | --- | --- |
|  |  | AA Transition^b^ | AA Transition^b^ |  |  | AA Transition^b^ | AA Transition^b^ |
| **PB2** | 9 | →D=D→N→D=D | →N=N=N→ND→DN | **PA(cont.)** | 332 | →S=S=S→PS→P | →P=P→PS=PS=PS |
|  | 22 | →R=R=R→KR→K | →K=K=K=K=K |  | 337 | →A=A=A=A=A | →S=S=S→SA→AS |
|  | 44 | →A=A=A=A=A | →SA→S=S→SA→AS |  | 352 | →K=K=K→EK→E | →E=E=E=E=E |
|  | 64 | →I=I=I→MI→M | →T=T=T→TM→MT |  | 354 | →V→I=I=I=I | →I=I=I=I=I |
|  | 66 | →T=T=T→MT→M | →M=M=M=M=M |  | 365 | →Q→P=P→Q=Q | →Q=Q=Q=Q=Q |
|  | 80 | →K→R=R→K=K | →K=K=K=K=K |  | 400 | →F=F=F→PL→P | →L=L=L→LP→PL |
|  | 81 | →T=T=T=T=T | →M=M=M→MT→T |  | 404 | →A=A=A=A=A | →SA→S=S→SA→AS |
|  | 105 | →T=T=T=T=T | →M=M→VM→VT→TV |  | 409 | →S→N=N→NS→N | →N=N=N=N=N |
|  | 106 | →T→A=A→TA→T | →T=T=T=T=T |  | 542 | →VI→I=I→VI→V | →V=V=V=V=V |
|  | 238 | →T→A=A→T=T | →T=T=T=T=T |  | 552 | →T=T=T=T=T | →S=S=S→ST→TS |
|  | 243 | →L=L=L→M=M | →M=M=M=M=M |  | 558 | →S→A=A→S=S | →S=S=S=S=S |
|  | 265 | →S=S=S→N=N | →N=N=N=N=N |  | 648 | →S→C=C→SC→S | →S=S=S=S=S |
|  | 271 | →T=T=T→TA→A | →A=A=A=A=A |  | 668 | →I=I=I=I=I | →IV→V=V→IV=IV |
|  | 340 | →K=K→R→RK→K | →R=R=R→KR→K |  | 689 | →S=S=S→AS→A | →A=A=A=A=A |
|  | 382 | →V=V=V→IV→I | →I→VI→V→IV=IV |  | **204** | →R=R=R=R→RK | →R=R=R→K=K |
|  | 444 | →V→IV→I→V=V | →V=V=V=V=V |  | **277** | →FS→F=F→SF→SH | →S→FS→F→YH→HY |
|  | 467 | →L=L=L→M=M | →M=M=M=M=M |  | **323** | →V=V=V→VI→IV | →V=V=V=V=V |
|  | 480 | →I=I=I→VI→V | →V=V=V=V=V |  | **356** | →R=R=R→KR=KR | →RK→R=R=R=R |
|  | 491 | →T=T=T=T=T | →A→T=T→TA→T | **NP** | 16 | →GD→G=G=G=G | →D=D=D→DG→GD |
|  | 508 | →R→Q=Q→RQ→R | →R=R=R=R=R |  | 21 | →N→D=D→ND→D | →N=N=N→ND→DN |
|  | 539 | →V=V=V→I=I | →I=I=I=I=I |  | 31 | →R=R=R=R=R | →K=K=K→KR→RK |
|  | 567 | →D=D=D=D=D | →N=N=N→ND→DN |  | 34 | →G=G=G=G=G | →DG→D=D→DG→GD |
|  | 588 | →A=A=A→AT→T | →I=I=I→IT→TI |  | 61 | →IL→I=I=I=I | →L=L=L→LI→IL |
|  | 613 | →V=V=V→VA→V | →TA→T=T→TV→V |  | 98 | →R=R=R→RK→R | →KR→K=K→RK→R |
|  | 649 | →V→I=I→VI→V | →V=V=V=V=V |  | 100 | →IV→I=I→VR→IV | →V=V=V→VI→IV |
|  | 661 | →A→G→S→A=A | →T=T=T→TA→AT |  | 109 | →I=I=I=I=I | →VI→V=V→VI→IV |
|  | 667 | →V=V=V→VI→IV | →VI→I=I→VI=VI |  | 119 | →I→V=V→IV→V | →I=I=I→IV→VI |
|  | 674 | →A=A=A=A=A | →T=T=T→TA→AT |  | 127 | →ED→E=E=E=E | →D=D=D→ED→E |
|  | 699 | →K→R=R→K=K | →K=K=K=K=K |  | 136 | →MI→I=I→IL→I | →M=M=M→IM→I |
|  | **54** | →K=K=K=K→KR | →K=K=K→KR→RK |  | 146 | →A=A=A=A=A | →TA→T=T→AT→A |
|  | **184** | →M=M=M→T→TA | →T=T=T→TA→AT |  | 189 | →M→I=I→IM→I | →M=M=M→MI→IM |
|  | **292** | →I→V=V→I→IV | →TI→T=T→TV→VT |  | 214 | →R=R=R=R=R | →K=K=K→KR→RK |
|  | **315** | →M=M=M=M→MI | →M=M=M→MI→IM |  | 217 | →I=I=I=I→VI | →I→S→GS→SV→VS |
|  | **453** | →P=P→S→PS=PS | →HP→H=H→HS→SH |  | 283 | →LP→L=L=L=L | →P=P=P→PL→LP |
|  | **559** | →T=T=T→TS→SI | →T=T=T→IAT→IA |  | 286 | →A=A=A=A=A | →A=A=A→SA→AS |
|  | **560** | →V=V=V→VL→LV | →V=V=V=V=V |  | 289 | →HY→H=H→YH→H | →Y=Y=Y→YH→HY |
|  | **684** | →T=T→A=A→AS | →AS→S=S→SA→S |  | 293 | →R=R=R=R=R | →KR→K=K→KR→RK |
| **PB1** | 54 | →K=K=K=K=K | →RK→K=K→KR→K |  | 305 | →R→K=K→KR→K | →KR→K=K=K=K |
|  | 80 | →S→T=T→S=S | →S=S=S=S=S |  | 334 | →H=H=H=H=H | →NH→N→NH→H=H |
|  | 97 | →K→KR→K→EK→E | →E=E=E=E=E |  | 345 | →S→G=G→S=S | →S=S=S=S=S |
|  | 108 | →L=L=L=L=L | →IL→L=L→LI→L |  | 350 | →KT→K=K→TK→K | →T=T=T→TK→KT |
|  | 129 | →Y→F=F→Y=Y | →Y=Y=Y=Y=Y |  | 353 | →V=V=V→VI→I | →IV→S=S→SI→IS |
|  | 152 | →L=L=L→SL→S | →S=S=S=S=S |  | 371 | →M→V=V→MV→V | →M=M=M→MV→VM |
|  | 158 | →N→SN→S→N=N | →N=N=N=N=N |  | 372 | →E=E=E=E=E | →DE→D=D→DE→ED |
|  | 177 | →GE→E=E=E=E | →E=E=E=E=E |  | 375 | →DE→D=D=D=D | →E=E=E→GD→DG |
|  | 182 | →T→V=V→T=T | →T=T=T=T=T |  | 400 | →R→K=K→RK→K | →R=R=R→RK→KR |
|  | 211 | →RK→K=K→KR→RK | →R=R=R=R=R |  | 421 | →ED→E=E=E=E | →D=D=D→E=E |
|  | 212 | →L=L=L→LV=LV | →L→LV→V→LV=LV |  | 422 | →R=R=R=R=R | →KR→K=K→KR→RK |
|  | 218 | →L→I→LI→L=L | →L=L=L=L=L |  | 423 | →A=A=A=A=A | →TP→P→SP→SA→AS |
|  | 261 | →N→S=S=S=S | →S→NS→S=S=S |  | 425 | →VI→V=V→IV→V | →I=I=I→VI=VI |
|  | 327 | →R=R=R=R=R | →R→RK→K→KR→RK |  | 442 | →T=T=T=T=T | →AT→A=A→AT→TA |
|  | 336 | →V=V=V→VI→I | →V=V→IV→I=I |  | 455 | →D=D=D=D=D | →ED→E=E→ED→DE |
| **PB1(cont.)** | 361 | →S→N=N→SR→R | →S=S→RS=RS→R | **NP(cont.)** | 473 | →S→N=N→NS=NS | →NS→N=N=N=N |
|  | 368 | →V→I=I=I=I | →I=I=I=I=I |  | 482 | →S→N=N→S=S | →S=S=S=S=S |
|  | 374 | →S=S=S→A=A | →A=A=A=A=A |  | **53** | →E=E=E=E=E | →E=E=E→ED→DE |
|  | 375 | →SG→G=G→SN→S | →SN→S=S=S=S |  | **313** | →FY→F=F=F→FV | →Y=Y=Y→YV→VY |
|  | 379 | →K→R=R→K=K | →K=K=K=K=K | **M1** | 15 | →V→I→V→VI→IV | →V=V=V→VI→I |
|  | 383 | →D=D=D→E=E | →DE→E=E=E=E |  | 95 | →K=K→KR→RK=RK | →RK→R=R→RK→R |
|  | 400 | →TA→A=A→T=T | →T=T=T=T=T |  | 115 | →V→I→VI→V=V | →I=I=I→IV→V |
|  | 430 | →K→E=E→KR→K | →RK→KR=KR=KR→K |  | 116 | →A→S→A→AS→SA | →A=A=A→AS→S |
|  | 473 | →L=L=L→V=V | →LV→V=V→VL→V |  | 137 | →T→A→TA→T=T | →A=A=A→AT→T |
|  | 576 | →I=I=I→L=L | →IL→L=L→LI=LI |  | 167 | →T=T→TA→T=T | →T=T→AT→TA→T |
|  | 581 | →D=D=D→DE→D | →E=E=E→D=D |  | 218 | →T=T→TA→T=T | →AT→A=A→AT→T |
|  | 621 | →KQ→Q=Q→RQ→R | →Q=Q=Q→RQ→R |  | 231 | →D→N→D=D=D | →DN=DN=DN=DN→D |
|  | 645 | →V=V=V=V=V | →MV→V=V=V=V | **M2** | 11 | →TI→IT→I→TI=TI | →I=I=I→IT→TI |
|  | 648 | →A→S=S→AS→A | →A=A=A=A=A |  | 13 | →NS→S=S→N→SN | →N=N→NS=NS→SN |
|  | 654 | →T=T=T→S=S | →NS→S=S→SN→S |  | 14 | →E=E=E→GE→EG | →E=E=E=E=E |
|  | 691 | →K=K=K=K=K | →RK→K=K→KR→K |  | 16 | →G=G=G→EG→E | →G=G=G→GE→EG |
|  | 739 | →D=D=D→E=E | →E=E=E=E=E |  | 28 | →A=A→AV→I→ID | →VI→V=V→VI→IV |
|  | 741 | →A=A=A→AS→S | →TA→A→AS→S=S |  | 54 | →R=R=R=R=R | →LRF→F=F→LIR→RL |
|  | 752 | →D=D=D→E=E | →E=E=E=E=E |  | 55 | →L→F=F→FL→F | →F→FL→F=F=F |
|  | **12** | →V=V=V=V→VI | →V=V=V→VI→IV |  | 56 | →EK→K=K=K=K | →K→EK→E→K=K |
|  | **113** | →V=V=V→VI→IV | →V=V=V=V→VA |  | 57 | →Y=Y=Y=Y=Y | →HY→H=H→HY→YH |
|  | **175** | →D=D=D=D→DN | →DN→D=D→DN→ND |  | 78 | →Q=Q=Q=Q=Q | →K=K=K→KQE→QK |
|  | **216** | →S=S=S→SG=SG | →S=S=S→GS→G |  | 79 | →K=K=K→EK=EK | →E=E=E=E=E |
|  | **298** | →L=L=L=L→LI | →LI→L=L→IL=IL |  | 82 | →S→NS→S=S=S | →S=S=S→NS→SN |
|  | **364** | →L=L=L=L→LI | →L=L=L→LI→IL |  | 86 | →V=V=V=V=V | →A=A=A→AV→VA |
|  | **386** | →R=R=R=R→RK | →R=R=R→KR=KR |  | 89 | →G=G=G=G=G | →SG→S=S→SG→GS |
|  | **433** | →K=K=K→KR→RK | →K=K=K=K=K |  | 95 | →E→VE→AV→EV=EV | →E=E=E=E=E |
|  | **435** | →A=A→AT→T→TI | →T=T=T→TI→IT |  | *93* | →N=N=N=N=N | →SN→S=S→SN=SN |
|  | **517** | →I=I=I→IV=IV | →I=I=I→VI=VI | **NS1** | 3 | →SF=SF→S=S=S | →P→S=S=S=S |
|  | **587** | →A=A=A→AT→AV | →A=A=A→AV→V |  | 18 | →V→I=I→IV→I | →V=V=V→IV→I |
|  | **618** | →E→KT→K→E→ED | →E=E=E→ED→DE |  | 21 | →R=R=R=R=R | →R→Q=Q→QR→RQ |
|  | **642** | →N=N=N→NS→SN | →N=N=N=N=N |  | 22 | →F=F=F=F=F | →V=V=V→VF→FV |
|  | **728** | →I=I=I=I→IV | →I=I=I→IV→VI |  | 25 | →KN→N=N→NW→N | →Q=Q=Q→QN→NQ |
| **PB1-F2** | 34 | ==→N→S=S | ==→N=N→S |  | 26 | →K→GE→G=G=G | →E=E=E→EG→G |
|  | 47 | ==→N→NS→N | ==→S→SN→N |  | 44 | →R=R→K→KR→K | →R=R=R→KR=KR |
|  | 49 | ==→L→AV→V | ==→VA→V=V |  | 56 | →T=T=T→TP→T | →T→AT→A→TA=TA |
|  | 50 | ==→V→DG=DG | ==→D→DA→D |  | 59 | →R→L=L→LR→L | →R=R→HR→HLC→LH |
|  | 52 | ==→P→HL→H | ==→H→HP→H |  | 60 | →A=A=A→VA→V | →AV→V=V=V=V |
|  | 65 | ==→R→RK→K | ==→K=K=K |  | 67 | →R→W=W→WR→W | →R=R→KR→KWR→WK |
|  | 69 | ==→P→Q=Q | ==→Q=Q=Q |  | 70 | →E→KR→K→KE→K | →K=K=K=K=K |
|  | 73 | ==→K→KR→R | ==→K→R=R |  | 74 | →ND→N=N→DS→S | →D=D=D→DS→SD |
|  | 75 | ==→H→RH→H | ==→R→H=H |  | 75 | →GE→E=E=E→ED | →E=E=E=E=E |
|  | 76 | ==→V→VA→A | ==→AV→A=A |  | 76 | →A→TA→T→TA→T | →A=A=A→AT→TA |
|  | 84 | ==→S→N=N | ==→N=N=N |  | 81 | →I=I=I=I=I | →M=M=M→MI→IM |
|  | 86 | ==→H→Q=Q | ==→Q=Q=Q |  | 84 | →V=V=V=V=V | →VA→A→TA→TVA→VT |
|  | 87 | ==→EG=EG→G | ==→G=G=G |  | 86 | →A→S=S→TA→T | →A=A=A→AT→TA |
|  | 90 | ==→N=N=N | ==→D→N=N |  | 95 | →L=L=L=L=L | →LI→I=I→IL→LI |
|  | **4** | ==→E→EG→E | ==→E=E→GE |  | 98 | →M=M=M=M=M | →M→LM→L→ML=ML |
|  | **6** | ==→D=D=D | ==→D→GD=GD |  | 114 | →S=S=S→SP→P | →P=P=P=P=P |
|  | **20** | ==→K=K=K | ==→K→KR→RK |  | 116 | →CY→Y→C=C=C | →C=C=C=C=C |
|  | **21** | ==→K→KG→K | ==→K→R→G |  | 125 | →E=E=E→ED→E | →D=D=D→ED→E |
|  | **23** | ==→S→NS→N | ==→S→SD→SN |  | 145 | →I=I=I=I=I | →I=I=I→VIT→IV |
|  | **27** | ==→T=T→TI | ==→T→IT→I |  | 166 | →L=L=L=L=L | →L=L→FL=FL→LF |
|  | **29** | ==→R→RK→R | ==→RK→K→KR |  | 178 | →V=V=V→IV→VI | →IV→I=I→IV→VI |
|  | **37** | ==→Q→R=R | ==→Q→QR=QR |  | 179 | →G→E→G=G=G | →G=G=G=G=G |
|  | **44** | ==→R=R→K | ==→R→RK→R |  | 189 | →G=G=G→GD→G | →DN→D=D→DG→GD |
| **PB1-F2(cont.)** | **60** | ==→Q→QP→P | ==→Q→LP→L | **NS1(cont.)** | 196 | →E=E=E=E=E | →EK→K=K→EK=EK |
|  | **62** | ==→RH→L=L | ==→L→PL→P |  | 197 | →N=N=N→NA→N | →T=T→TN→NT→N |
|  | **70** | ==→AV→GE→G | ==→G→GV→V |  | 211 | →R=R=R=R=R | →R=R→RG→GR→RG |
|  | **71** | ==→F→Y=Y | ==→S=S=S |  | 213 | →S=S→P→SP→S | →P=P=P→PS→SP |
|  | **74** | ==→T→TI→I | ==→T=T=T |  | 215 | →P=P=P=P=P | →T=T=T→TP→PT |
|  | **82** | ==→L=L=L | ==→LS→SP→P |  | 217 | →K→E=E→EK→E | →K=K=K→KET→EK |
|  | **83** | ==→FS→SF=SF | ==→F=F=F |  | 220 | →W=W→RW→R=R | →R=R=R=R=R |
|  | **85** | ==→K=K=K | ==→K→KR→R |  | 222 | →V=V→VM→M=M | →M=M=M=M=M |
|  | **89** | ==→T→TI→I | ==→T=T=T |  | 227 | →R→G→GR→G=G | →R=R=R=R=R |
| **PA** | 20 | →AT→T=T→AT→A | →A=A=A=A=A |  | 228 | →S=S=S→PS→P | →S=S=S=S=S |
|  | 27 | →D→N=N→D=D | →D=D=D=D=D |  | **6** | →V=V=V=V→VM | →V=V=V→VM→MV |
|  | 28 | →P=P=P=P→PS | →L=L=L→LP→PL |  | **78** | →K=K=K=K→KR | →K=K=K→KR→RK |
|  | 42 | →M=M=M→LM→L | →L=L=L=L=L |  | **119** | →M=M=M=M→ML | →M→IM→I→ML→LM |
|  | 57 | →R=R=R=R→RQ | →Q=Q=Q→QR→RQ |  | **123** | →I=I=I=I→IV | →I=I=I→IV→VI |
|  | 65 | →S→P=P→SP→S | →L=L=L→LS→SL |  | **129** | →I=I=I→IT→IV | →I→MI→M→MVI→V |
|  | 66 | →G=G=G=G=G | →DG→D=D→DG→GD |  | **171** | →D=D=D→DN→NY | →NDA→IN→I→IYN→YI |
|  | 68 | →P→S=S→PS→P | →P=P=P=P=P |  | **194** | →V=V=V→VG→GV | →V=V=V=V=V |
|  | 85 | →TA→N=N→TN→TI | →T=T=T→TI→IT |  | **198** | →L=L=L=L→LI | →L=L=L→LI→IL |
|  | 100 | →V=V=V=V=V | →A=A=A→AV→VA |  | **206** | →R=R=R→RI→RC | →S=S=S→SC→CS |
|  | 101 | →G=G=G→E=E | →E=E=E=E→EG |  | **207** | →N=N=N=N→ND | →N→ND→N→ND→DN |
|  | 104 | →K→RK→R→KR→K | →K=K=K=K=K |  | **209** | →N=N=N→DN=DN | →N=N=N=N=N |
|  | 115 | →D=D=D→N=N | →N=N=N=N=N |  | *91* | →AT→A=A→AT→AS | →T=T=T→TS→ST |
|  | 142 | →E=E=E→K=K | →K→NK→N→KN=KN | **NS2** | 3 | →SF→FS→S=S=S | →P→SP→S=S=S |
|  | 208 | →T=T→K→KT→T | →T=T=T→TS→T |  | 14 | →M=M=M=M=M | →M→LM→L→ML=ML |
|  | 225 | →S=S=S=S=S | →CS→C=C→CS→SC |  | 22 | →G→R→G=G=G | →G=G=G=G=G |
|  | 227 | →E→DE→D→ED→E | →E=E=E=E=E |  | 27 | →G=G=G→DG→D | →D=D=D=D=D |
|  | 251 | →K→R=R→K=K | →K=K=K=K=K |  | 32 | →V=V=V→VI→V | →I=I=I→IV→VI |
|  | 268 | →L=L=L=L=L | →IL→I=I→IL→LI |  | 34 | →R→Q=Q→RQ→R | →Q=Q=Q→QR→RQ |
|  | 272 | →GC→C=C→D=D | →D=D=D→ND→DN |  | 40 | →I=I=I→IL→I | →L=L=L→IL→I |
|  | 291 | →S→N→S=S=S | →S=S=S=S=S |  | 57 | →Y=Y=Y→YS→Y | →S=S→LS→YLS→YL |
|  | 308 | →I→V=V→IV→I | →I=I=I=I=I |  | 63 | →G→EG→E→EG→E | →G=G=G→GE→EG |
|  | 309 | →K→R=R→K=K | →K=K=K=K=K |  | 86 | →R→K=K→RK→R | →KR→RK→R→RK→R |
|  | 312 | →K=K=K=K=K | →R=R=R→KR→K |  | 89 | →I=I→M→IA→A | →I→TI→T→TA→AT |
|  | 321 | →I→T=T→N=N | →YN→Y=Y→YN→NKY |  | 107 | →L=L=L=L=L | →FL→F=F→FL→LF |
|  | 322 | →I=I=I=I=I | →V→IV→I=I=I |  | 113 | →I→M=M→I=I | →I=I=I=I=I |

^a^The position number in regular face indicates this site was a signature within specific periods before 2010, but turned to a non-signature during 2010~2013. The number in boldface indicates this site was a nonsignature within specific periods before 2010, but became a signature during 2010~2013. The position number in italic indicates this site was a chronological signature throughout all periods, but their dominant AA varied.

^b^Transition of dominant amino acid residues on characteristic sites. Distinct dominant AA between different hosts are underlined. The symbol “→” indicates a transition of dominant amino acids between two periods, e.g. VM→VT or DE→D; the symbol “=” indicates there was no change in dominant amino acids, e.g. T=T or A=A. Because of the lack of protein data, we did not show the chronological signatures in some periods, as denoted by .

**Table S6. Top 20 sites in PB2 and their amino acid residues**

| **ARI** | | | | **MI** | | | |
| --- | --- | --- | --- | --- | --- | --- | --- |
| **Position** | **Value** | **Avian** | **Human** | **Position** | **Value** | **Avian** | **Human** |
|  |  | **AA (percent)^a^** | **AA (percent)^a^** |  |  | **AA (percent)^a^** | **AA (percent)^a^** |
| 271 | 0.798027 | T (97%) | A (99%) | 271 | 0.871188 | T (97%) | A (99%) |
| 588 | 0.629986 | A (95%) | IT (57% 42%) | 588 | 0.847819 | A (95%) | IT (57% 42%) |
| 684 | 0.595872 | A (98%) | S (81%) | 292 | 0.613760 | I (86%) | TV (55% 40%) |
| 453 | 0.444679 | P (92%) | SH (44% 43%) | 684 | 0.596938 | A (98%) | S (81%) |
| 292 | 0.439519 | I (86%) | TV (55% 40%) | 453 | 0.572104 | P (92%) | SH (44% 43%) |
| 475 | 0.292138 | L (99%) | ML (57% 42%) | 559 | 0.350855 | T (91%) | IT (42% 28%) |
| 559 | 0.280201 | T (91%) | IT (42%28%) | 567 | 0.345579 | D (94%) | ND (55% 43%) |
| 627 | 0.273292 | E (98%) | KE (56% 44%) | 475 | 0.336871 | L (99%) | ML (57% 42%) |
| 368 | 0.270795 | R (98%) | KR (56% 43%) | 105 | 0.336016 | T (97%) | VT (53% 43%) |
| 567 | 0.259921 | D (94%) | ND (55% 43%) | 627 | 0.333002 | E (98%) | KE (56% 44%) |
| 613 | 0.255608 | V (97%) | TV (52% 44%) | 199 | 0.330784 | A (99%) | SA (57% 43%) |
| 199 | 0.250793 | A (99%) | SA (57% 43%) | **81^b^** | 0.329765 | **T** (99%) | **M**T (43% 43%) |
| 674 | 0.245027 | A (94%) | TA (55% 44%) | 613 | 0.326935 | V (97%) | TV (52% 44%) |
| 702 | 0.237781 | K (96%) | RK (56% 44%) | 368 | 0.322030 | R (98%) | KR (56% 43%) |
| 64 | 0.232468 | M (98%) | TM (56% 43%) | 64 | 0.310181 | M (98%) | TM (56% 43%) |
| 44 | 0.230203 | A (99%) | SA (56% 44%) | 674 | 0.303785 | A (94%) | TA (55% 44%) |
| 105 | 0.222788 | T (97%) | VT (53% 43%) | 9 | 0.301805 | D (97%) | ND (53% 43%) |
| 661 | 0.213315 | A (92%) | TA (55% 44%) | 44 | 0.293692 | A (99%) | SA (56% 44%) |
| 590 | 0.197782 | G (84%) | SG (61% 38%) | **645^b^** | 0.254643 | **M** (100%) | **M**L (56% 44%) |
| 9 | 0.194888 | D (97%) | ND (53% 43%) | **591^b^** | 0.253886 | **Q** (98%) | **Q**R (56% 43%) |

^a^We showed only the dominant residues with more than 20% conservation.

^b^At positions 645, and 591, the most dominant residues, in boldface, are the same in avian and human. Though the most dominant residues at position 81 are not the same in avian and human (T vs. M), the difference between T’s and M’s conservations on human PB-2 is marginal (M=42.98% vs. T= 42.77%). None of these sites is an appropriate signature.
